# PPARγ in Ischemia-Reperfusion Injury: Overview of the Biology and Therapy

**DOI:** 10.3389/fphar.2021.600618

**Published:** 2021-04-28

**Authors:** Ruizhen Huang, Chiyu Zhang, Xing Wang, Honglin Hu

**Affiliations:** Department of Urology, The Second Affiliated Hospital of Nanchang University, Nanchang, China

**Keywords:** PPARγ, ischemia-reperfusion injury, Mechanisms, Therapeutic potential, Protective effect

## Abstract

Ischemia-reperfusion injury (IRI) is a complex pathophysiological process that is often characterized as a blood circulation disorder caused due to various factors (such as traumatic shock, surgery, organ transplantation, burn, and thrombus). Severe metabolic dysregulation and tissue structure destruction are observed upon restoration of blood flow to the ischemic tissue. Theoretically, IRI can occur in various tissues and organs, including the kidney, liver, myocardium, and brain, among others. The advances made in research regarding restoring tissue perfusion in ischemic areas have been inadequate with regard to decreasing the mortality and infarct size associated with IRI. Hence, the clinical treatment of patients with severe IRI remains a thorny issue. Peroxisome proliferator-activated receptor *γ* (PPARγ) is a member of a superfamily of nuclear transcription factors activated by agonists and is a promising therapeutic target for ameliorating IRI. Therefore, this review focuses on the role of PPARγ in IRI. The protective effects of PPARγ, such as attenuating oxidative stress, inhibiting inflammatory responses, and antagonizing apoptosis, are described, envisaging certain therapeutic perspectives.

## Introduction

Good blood circulation in organs is the premise for their normal physiological function. Ischemic diseases are common in clinical practice; however, they sometimes cause severe damage to organs if not considered seriously. The development of ischemic diseases mainly consists of the following two stages: ischemia and reperfusion. Blood circulation under normoxic conditions guarantees tissue physiological function. Paradoxically, many studies have revealed that when the blood flow is restored in ischemic tissue, it exhibits severe metabolic dysregulation and structural destruction. This phenomenon is known as ischemia-reperfusion injury (IRI).

Complex molecular mechanisms and deterioration of cell microstructure are important factors contributing to organ IRI, thereby damaging the body seriously. Since IRI is a multifactorial disease, the divergent target organs undergoing damage pose a difficult challenge during clinical therapy. With an increasing number of studies in this area, researchers have gradually found that the main factor causing damage to the tissue is not ischemia itself, but rather the excessive free radicals generated by cells after restoration of blood supply, which attack the part of the tissue that has reacquired blood supply. The mechanisms of IRI are complicated and remain largely unclear. Many studies have reported that the production of reactive oxygen species (ROS), apoptosis, necrosis, and inflammatory cell infiltration are the main cellular responses in the pathophysiological process of IRI, subsequently leading to tissue damage due to a series of continuous cell events (Friedewald and Rabb, 2004; Kieran and Rabb, 2004).

The PPAR family consists of three isoforms, namely, PPARα, PPARβ/δ, and PPARγ, which control gene expression in downstream networks to participate in lipogenesis, lipid metabolism, inflammation, and maintenance of metabolic homeostasis. In addition, certain other effects of PPAR activation, such as tumor-inhibitory effects, have been identified in most experimental models of PPARs. The mechanism may be related to the suppression of proliferation as well as induction of differentiation and apoptosis (Evans et al., 2004; Barish et al., 2006). The potential adverse consequences of PPAR activation also need to be carefully considered. For instance, GW501516 (a type of PPAR-δ synthetic agonist) may accelerate the growth of small intestinal polyps in adenomatous polyposis *coli* tumor suppressor gene-deficient mice. The occurrence and size of colon polyps are augmented by PPARγ agonists as well (Lefebvre et al., 1998; Gupta et al., 2004).

Activation of PPARγ can lead to key consequences in mitochondrial metabolisms of fatty acid (FA) and glucose. Excess lipid accumulation is associated with decreased organ function. It may be due to either toxic effect of intracellular lipids or excessive fatty acid oxidation (FAO). PPARγ can overexpress FAO genes, and greater FAO is associated with more ROS production (Bhaumik et al., 2005). It can also deviate FA to the toxic ceramide and DAG; however, despite increased DAG and ceramide levels, research showed that partitioning of lipid to storage and oxidation could reverse cardiolipotoxicity in PPARα-deficiency mice, reflecting the intricate metabolic regulation of PPARγ (Son et al., 2010).

PPARγ is subdivided into γ1 and γ2. PPARγ1 is expressed in a wide range of tissues, such as the myocardium of mammals, skeletal muscle, colon, small and large intestines, kidney, pancreas, and spleen, while PPARγ2 is primarily expressed in adipose tissue (Fajas et al., 1997), which confirms the subsequent finding that PPARγ activation reduces IRI. Studies have shown that PPARγ1 and 2 are involved in immune regulation, regulation of lipid metabolism, and inflammation, and are thus associated with the underlying molecular mechanism of IRI. It has been reported to play an important role in the reduction of anti-inflammatory cytokine biosynthesis (Haraguchi et al., 2008) and in the improvement of cellular antioxidant systems (Shimazu et al., 2005). The anti-inflammatory role of PPARγ in myeloid-lineage cells has been reported to be important in controlling pro-inflammatory cytokine synthesis, myeloid-derived suppressor cell expansion, immunosuppression, and cancer development (Wu et al., 2012). In addition, it has been revealed that PPARγ plays an important role in organ IRI (Cuzzocrea, 2004). Protective effects of PPARγ agonists have been reported in cerebral, cardiac, kidney, and hepatic IRI. Activation of PPARγ mainly depends on its natural ligand and synthetic ligand. Thiazolidinediones (TZDs) such as rosiglitazone (RGZ) and pioglitazone (PIO), which are synthetic ligands of PPARγ, have been reported to exhibit significant improvements in antidiabetic therapy (Murphy and Holder, 2000). In addition, the transcription of target genes related to lipid metabolism, glucose homeostasis, cell proliferation, and differentiation, as well as inflammation, has been confirmed to be altered by TZDs (Chinetti et al., 2003). Growing evidence indicates that the therapeutic effects of PPARγ ligands may also be applied to other diseases.

## The Potential Molecular Mechanisms of Ischemia-Reperfusion Injury

The pathological mechanism of IRI is complex and involves many biological processes. The mechanism of its occurrence has not been fully elucidated. Importantly, it is currently believed that increased free radical production, intracellular calcium overload, and excessive activation of inflammatory response are essentially involved in IRI. The microvascular and parenchymal organ damage induced by ischemia tissue reperfusion is mainly caused by reactive oxygen–free radicals and has been demonstrated in many organs. The synthesis of antioxidant enzymes that scavenge free radicals in ischemic tissue is then impaired, thus exacerbating the damage caused by free radicals to postischemic reperfusion tissue. Free radical scavenging by superoxide dismutase (SOD) protects against IRI.

Clinicians are beginning to pay attention to the benefits of using free radical scavengers such as antioxidants to reduce the harmful effects of excessive free radicals while restoring blood and oxygen supply to ischemic tissues. Here, we provide an overview of the underlying molecular mechanism involved in promoting these abovementioned effects that eventually result in IRI.

### Free Radicals

Free radicals are important oxygen metabolites and are mainly divided into two categories, namely, ROS and reactive nitrogen species (RNS). Due to their active chemical properties, they interact with antioxidants and are destroyed, while excessive amounts will cause oxidative stress such as lipid peroxidation in the body, destruction of protein and nucleic acid structure and function, and tissue damage (Chouchani et al., 2014; Phull et al., 2018). Two types of important chemical substances in the body play protective roles, such as antioxidants (glutathione, vitamin C, coenzyme Q, and so on) and antioxidant enzymes, such as SOD, catalase, and glutathione peroxidase (GSH-Px), and can remove free radicals in a timely manner and thereby maintain normal physiological functions of the body.

During ischemia, tissue cells are hypoxic. As a result, partial pressure of intracellular oxygen decreases, leading to dysfunctional mitochondrial oxidative phosphorylation, decreased ATP production, increased calcium entry into the mitochondria, dysfunction of the cytochrome oxidase system, and damage to the electron transport chain. The activity of antioxidant enzymes such as catalase, GSH-Px, and SOD decreases. When the tissue is restored with blood flow, the oxygen entering the cells increases through the mitochondria to form active oxygen species, which induces oxidative stress (Inceoglu et al., 2017; Garcia et al., 2018; Yu et al., 2019).

Neutrophils play a vital role in maintaining homeostasis. The oxygen consumption during phagocytosis is significantly increased, and the formation of free radicals can kill pathogenic microorganisms. Free radicals generated during ischemia act on cell membranes, and the production of leukotrienes can attract a large number of neutrophils and activate them. When the tissue regains oxygen, the neutrophils consume a lot of oxygen and produce many free radicals, causing tissue damage (Kalogeris et al., 2012).

Studies have shown that the metabolism of uric acid mediated by xanthine oxidase and its precursor xanthine dehydrogenase is closely related to the generation of free radicals. ATP levels seldom affect uric acid metabolism during hypoxia (Pelin et al., 2018). During reperfusion, increased amount of dioxygen enters the ischemic tissue with the blood, and the metabolism of uric acid is restored. The accumulation of active oxygen generated during metabolic restoration of uric acid is the precise mechanism leading to tissue damage (Watanabe et al., 2019). In addition, during ischemia-reperfusion, although the sympathoadrenal medulla system can be stimulated to produce catecholamines in response to stress, it is also accompanied by the production of free radicals. An imbalance between increased oxidative substances and reduced antioxidant defense mechanisms leads to oxidative stress–induced damage.

### Calcium Overload

Under physiological conditions, there is a huge difference between intracellular and extracellular calcium concentration. This difference in concentration is sustained through a series of transport mechanisms that maintain a dynamic balance so that all kinds of biochemical reactions can be carried out normally. Ischemia-reperfusion may cause abnormal calcium transport, thereby increasing intracellular calcium content, which leads to calcium overload, cell structure destruction, and metabolic dysfunction. Calcium overload may aggravate body damage through energy metabolism disorders, cell membrane and structural protein breakdown, and aggravation of acidosis. In particular, mitochondrial calcium overload is a crucial event in determining the fate of cell survival and death (Ben-Ari et al., 2009; Lee et al., 2018). However, the mechanism of calcium overload caused by ischemia-reperfusion has not been fully studied. Possible factors include abnormal Na^+^–Ca^2+^ exchange, protein kinase C (PKC) activation, and biofilm damage.

### Excessive Activation of Inflammation

It is acknowledged that ischemia-reperfusion can activate aseptic inflammatory response in the body, mainly involving the aggregation and activation of immune cells and activation of the complement system. Among them, leukocyte aggregation and activation-mediated microvascular injury play important roles in organ IRI (Kurose et al., 1994). Activated neutrophils and vascular endothelial cells release large amounts of active substances, such as free radicals, proteases, and lysozymes, resulting in damage to peripheral tissue cells. At the same time, aggregated granulocytes adhere to vascular endothelial cells and block microcirculation in vessels. Ischemic hypoxia induces a series of cascade reactions that further aggravate tissue hypoxia.

Ischemic injury stimulates the increased expression of various adhesion molecules on the surface of vascular endothelial cells, which mainly include integrins, selectins, and intercellular adhesion molecules. Chemokines, selectins, and integrins secreted by endothelial cells and leukocytes can promote the exudation of neutrophils and macrophages, as well as increase in cytokines such as leukotrienes (LTs), leukotriene B4 (LTB4), and platelet activating factor (PAF). Moreover, other factors released during inflammatory reaction can attract a large number of white blood cells to adhere to the vascular endothelium or exudate, thereby causing tissue damage (Cannistra et al., 2016).

### Other Important Biological Processes

The process of cell apoptosis has been implicated as an eminent symptom of IRI; it can be roughly divided into the following stages: receiving apoptotic signals, interactions between apoptosis regulatory molecules, activation of proteolytic enzymes (caspase), and the cascade reaction process. After a proapoptotic stimulus signal, caspase precursors of initiator caspases were activated as a result of proteolytic processing. Once activated, initiator caspases could activate other caspases in a cascade, leading to the activation of effector caspases; a variety of proteins were subsequently degraded, and apoptosis process was initiated simultaneously (Fleisher, 1997).

By recycling damaged and toxic cytoplasmic material into new cellular building blocks, autophagy supports anti-stress responses and energy maintenance. The damaged proteins and organelles were disposed of by autophagy as well, so it plays an indispensable role in the face of starvation and other kinds of stress (Glick et al., 2010). Published works showed contradictory findings; both protective and detrimental roles of autophagy were manifested. Autophagy was also suggested to provoke cell death; it could be a double-edged sword in terms of its role in cerebral, kidney, liver, and myocardium reperfusion injury, although the mechanisms behind it have been a matter of debate (Rautou et al., 2010; Puyal et al., 2012; Decuypere et al., 2015; Sciarretta et al., 2018).

N-methyl-D-aspartate (NMDA) is the excitatory neurotransmitter involved in learning and memory. During the ischemia period, glutamate accumulated rapidly at synapses in the brain, resulting in extensive stimulation of its N-methyl-D-aspartate receptors (NMDARs), which could be toxic to neurons eventually (Simon et al., 1984; Rossi et al., 2000). Excessive and prolonged activation of NMDARs induced a large amount of Ca^2+^ influx through the cytomembrane. Intracellular Ca^2+^ overloaded could subsequently trigger several downstream lethal reactions including proteolysis of key cellular proteins, resulting in irreversible cell death (Lipton, 2006). Thus, NMDAR excitotoxicity results in the severe cell metabolic disorder. NMDARs have long been considered as a therapeutic target for IRI insults. Recently, the role of NMDA was also discovered in peripheral organs such as bones, cardiovascular, and kidneys (Bozic and Valdivielso, 2015).

## Role of Peroxisome Proliferator-Activated Receptor *γ* in Renal Ischemia-Reperfusion Injury

The kidney is a highly perfused organ that is sensitive to ischemia and reperfusion injury, and the incidence of renal IRI has high morbidity and mortality. Previous reports have attributed tissue damage during IRI to be associated with ROS generation, apoptosis, necrosis, infiltration of inflammatory cells, and release of active mediators (Friedewald and Rabb, 2004; Kieran and Rabb, 2004). Several studies have shown that apoptosis and oxidative stress are important mechanisms involved in renal IRI. Over the years, it is increasingly believed that the main form of cell death during IRI is apoptosis, and it is worth noting that it can even affect the outcome of cells in an inflammation-independent manner (Ysebaert et al., 2004; Ballow et al., 2005; Docherty et al., 2006). Although PPARγ is abundantly expressed in adipose tissue, it is also expressed in vascular smooth muscle cells, endothelial cells, monocytes, macrophages, and myocytes. Indeed, ischemia induces cell apoptosis in various segments of renal tubules (Okada et al., 1997; Xu et al., 2006). In our previous studies, pretreatment of mice, subjected to ischemia-reperfusion, with the PPARγ agonist pioglitazone resulted in mitigating IRI-induced renal cell apoptosis, with an increase in Bcl-2 expression and a decrease in Bax expression (Hu et al., 2012). As mentioned above, the overproduction of ROS after tissue reperfusion has long been recognized as one of the key mechanisms that trigger cell apoptosis. Furthermore, we also indicated that the renoprotective properties of pioglitazone might be attributable in part to enhancing the antioxidant capacity of the kidney (Zou et al., 2013); it may be recognized as one of the pivotal mechanisms implicated in the anti-inflammatory effects of PPARγ.

During IRI, cells induce autophagy (Ma et al., 2015). Understanding the process of autophagy has been complicated for a long time. It has been reported that when the autophagy-related gene ATG5-deficient mice were used to establish an IRI model, a dramatic increase in serum creatinine and urea nitrogen was detected, together with an increased rate of apoptosis, suggesting a significant protective effect of autophagy against IRI (Kimura et al., 2011). Multiple signaling molecules, including the mammalian target of rapamycin (mTOR), the major inhibitory signal of autophagy, AMP-activated protein kinase (AMPK), Bcl-2/beclin 1 complex, and p53, play a crucial part in the regulation of autophagy. The class I phosphatidylinositol 3-kinase (PI3K)/Akt and p70S6 kinase are linear substrates of mTOR that may act to regulate mTOR activity, either by inhibiting or enhancing it. AMPK is another sensor of cellular bioenergetics, especially in response to energy stress (Yang and Klionsky, 2010). In our subsequent study, a pivotal renoprotective role of the AMPK–mTOR pathway in enhancing autophagy induced by PIO was presented, by examining the total upregulation of AMPK phosphorylation. However, this effect was suppressed upon administration of the PPAR-γ inhibitor GW9662 and autophagy inhibitor 3-MA (Chen et al., 2018). In addition, it has been reported that PIO could significantly reduce the rate of apoptosis (Reel et al., 2013). GW9662 and 3-MA reversed this effect of PIO, thereby providing further evidence that PIO reduces IRI-induced apoptosis.

Previously, it has been reported that in MCF-7 cells (human breast cancer cells), PIO treatment could enhance PPARγ transcriptional activity (Sato et al., 2013). A murine model of intestinal IRI showed that PPARγ-deficient mice exhibited more severe kidney damage; however, that adverse impact on tissues could be abrogated by the activation of PPARγ (Nakajima et al., 2001). Similarly, PPARγ-deficient mice that developed liver IR showed worse liver function after IR than wild-type (Wt) mice. However, PPARγ agonists reduced the levels of ALT and AST, thereby reflecting alleviation of liver injury in Wt mice (Kuboki et al., 2008). After IR, the expression of liver PPARγ was up-regulated and several strong pro-inflammatory cytokines, chemokines, and adhesion molecules were down-regulated by PPARγ agonist administration, thereby inhibiting IRI (Akahori et al., 2007). The specific targets of PIO involved in protecting organ IRI still need further exploration. Further research is required to understand the molecular mechanisms of PPARγ activation involved in protecting against renal IRI.

The most widely studied cyclopentenone prostaglandin, 15-deoxy-Δ12,14-prostaglandin J2 (15 days-PGJ2), is considered as the first natural ligand of PPARγ, and exhibits anticancer, anti-inflammatory (Bianchi et al., 2014), antioxidative (Ruiz-Miyazawa et al., 2018), and protective effects during ischemia-reperfusion in animal models (Chen K. et al., 2017). Whether its anticancer and anti-inflammatory effects rely on the activation of PPARγ remains to be investigated. It was first demonstrated that the protective effects of 15 days-PGJ2 in acute severe hemorrhage and resuscitation were primarily an outcome of PPARγ activation (Abdelrahman et al., 2004). Several studies have been implemented to investigate the effects of 15 days-PGJ2 on tissue injury resulting from IR *in vitro* and *in vivo*. Hypoxia diminished the expression and anti-inflammatory effects of PPARγ in human proximal renal tubular epithelial cells (HPTECs) (Li et al., 2007). Lin et al. reported that upregulation of the protective heme oxygenase-1(HO-1) combined with inhibition of COX-2 and other NF-κB–dependent genes upon PPARγ activation were the primary mechanisms by which 15 days-PGJ2 and rosiglitazone prevent IR-induced neuronal necrosis and apoptosis, thereby limiting infarction area expansion. The use of two kinds of PPARγ inhibitors, including GW9662 and BADGE, further illustrated that the protective effects of 15 days-PGJ2 were mediated by PPARγ (Lin et al., 2006). In one experiment, the sensitivity of 15 days-PGJ2 to TNF-related ligand-induced apoptosis was not completely blocked by GW9662, suggesting a partial PPARγ-dependent influence of this ligand (Han et al., 2007). However, the specific mechanism by which 15 days-PGJ2 regulates PPARγ has not been fully elucidated. It may enhance PPARγ activity at the transcriptional level, or alternatively, it might be involved at the protein stability level. However, PPARγ ligands can alleviate tissue damage to a certain extent, and PPARγ may be a potential target for the clinical treatment of organ IRI. The protective effects of PPARγ in renal IRI are summarized in Table 1.

**TABLE 1:** Summarization of the effects of drugs acting on PPARγ in renal IRI.

Drugs act on PPARγ	IRI models	Main effects	Conclusion	Reference
Pioglitazone^a^	Male Wistar albino rats (200–250 g)	Antioxidant effects and renoprotection	NMDA receptor antagonism involves in pio-mediated renoprotection	Singh et al. (2016)
Pioglitazone^a^	NRK-52e cells	Inhibited oxidative stress and ERS	Pioglitazone can inhibit oxidative stress and ERS in RTECs under NG and HG conditions	Zou et al. (2019)
Pioglitazone^a^	Male Sprague–Dawley rats (200–250 g)	Inhibited apoptosis and exhibiting an antioxidant effect	Pioglitazone protects renal against IRI; AMPK and autophagy-related signals are engaged in	Chen et al. (2018)
Pioglitazone^a^	NRK-52e cells	Enhanced autophagy	Pioglitazone enhances AMPK phosphorylation and inhibits mTOR phosphorylation during IRI	Xi et al. (2019)
Rosiglitazone^a^	Male Sprague–Dawley rats (200–250 g)	Reduced inflammatory and apoptotic markers	RGZ-induced renoprotection is linked to a reduction of inflammatory and apoptotic markers, besides reversing the eNOS mRNA and iNOS mRNA expression	Betz et al. (2012)
Rosiglitazone and ciglitazone^a^	Wistar rats	Reduced oxidative stress and anti-inflammatory cytokines	Renoprotective effects of rosiglitazone and ciglitazone may *via* ICAM-1and PMN-relevant oxidative stress reduction	Sivarajah et al. (2003)
15 days-PGJ2^b^	Wistar rats weighing 215–305 g	Reduced pro-inflammatory gene expression	15 days-PGJ2 protects the kidney by reducing pro-inflammatory gene expression and inhibiting NF-κB activation	Chatterjee et al. (2004)
Cilostazol^c^	Adult male Wistar rats weighing 200–250 g	Modulated the oxidative stress, iNOS, NF-kB, IL-18, caspase-1, NGAL, Kim-1, and PPARγ level	Cilostazol purveys renoprotective effects may partially *via* the upregulation of PPARγ	Ragab et al. (2014)
Sildenafil^c^	Male Wistar albino rats weighing 200–250 g	Antioxidant and renoprotective effects	Sildenafil protects against IR-induced AKI through PPARγ agonism in rats	Mohey et al. (2016)
Estradiol^c^	Male Wistar albino rats (16–20 weeks, 200–250 g)	Upregulation, antioxidant, and antiapoptotic activity	Estradiol protects renal *via* PPAR-γ–stimulated eNOS activation	Singh et al. (2019)

NMDA, N-methyl-D-aspartic acid; RTECs, rat renal tubular epithelial cells; NG, normal glucose; HG, high glucose; ERS, endoplasmic reticulum stress; eNOS, endothelial NO synthase; iNOS, inducible NO synthase; PMN, polymorphonuclear; NGAL, neutrophil gelatinase-associated lipocalin.

a PPARγ synthetic ligand.

b PPARγ natural ligand.

c PPARγ agonist.

## Relationship Between Peroxisome Proliferator-Activated Receptor *γ* and Hepatic Ischemia-Reperfusion Injury

The occurrence of hepatic IRI may be due to network-type or burst-type inflammation. Studies have shown that treatment with PPARγ agonists such as 15 days-PGJ2 and rosiglitazone could regulate the production of pro-inflammatory mediators by Kupffer cells (Kuboki et al., 2008). Kupffer cells (KCs) are macrophages which are mainly located in the sinusoids of the liver. When activated, they can release various pro-inflammatory cytokines, such as TNF-α and IL-1β, in addition to releasing oxygen-free radicals. Accumulation of neutrophil granulocytes damages liver tissues, and the application of Kupffer cell–activating inhibitors can significantly reduce liver IRI (Kobayashi et al., 2002). To further investigate the relationship between PPARγ activation and KC polarization as well as IR liver lesions, PPARγ agonists and antagonists were administered to a nonlethal segmental (70%) hepatic warm ischemia mouse model. Upon activation of PPARγ, the pro-inflammatory nitric oxide^+^ Kupffer cell population decreased and the anti-inflammatory CD206^+^ Kupffer cell population increased in response to IRI; hepatic IRI was ameliorated. However, the PPARγ antagonist reversed this beneficial effect on liver lesions. Experimental results showed that the rate of hepatocellular apoptosis as well as the pro-inflammatory NO^+^ Kupffer cell population was increased. They concluded that amelioration of liver IRI was coupled with PPARγ activation and modification of tissue-resident macrophage polarization (Linares et al., 2018). Subsequently, another study also confirmed the protective effects of PPARγ activation in the liver due to modification of KC polarization. It has been reported that curcumin activates PPARγ and improves the decreased liver function caused by orthotopic liver transplantation by modifying the polarization of Kupffer cells from pro-inflammatory polarization (M1 KCs) to anti-inflammatory polarization (M2 KCs) (Liu et al., 2018). This further indicates that curcumin can protect mice against IRI and may be an important immune supplement therapy to improve clinical liver IRI outcome.

The important role of PPARγ in hepatic IRI has been acknowledged for a long time. However, the molecular mechanism of its protective effect in liver IRI is still unclear, thus making it an interesting research topic for several research groups. It has been reported that by activating the PPARγ/NLRP3 inflammasome signaling pathway in mice, asiatic acid (AA) dramatically alleviated hepatic damage post-IR (Xu et al., 2017). In steatotic livers, inhibition of angiotensin II action protects rats against IRI. By stimulating the production of bradykinin (BK), the adverse effects of IRI could be reversed. See the summarization in Table 2. It was further shown that BK exerts these protective effects on hepatic IRI through the PPARγ pathway (Casillas-Ramirez et al., 2008). Koh et al. reported that losartan could protect the hepatic cells from IR damage by activating PPARγ, which subsequently inhibited the RAGE-mediated signaling pathways, such as the MAPK and Egr-1 pathways. In addition to PPARγ, RAS inhibition or bradykinin B2 receptor agonist may participate in the hepatoprotective effects provided by losartan against IRI (Bonde et al., 2011; Koh et al., 2013). Recently, Ji and colleagues administered cafestol in mice before surgery-induced hepatic IRI and found that it eventually attenuated liver IR-induced apoptosis and autophagy by inhibiting the extracellular signal-regulated kinase (ERK)/PPARγ pathway (Ji et al., 2020). As the natural ligand of PPARγ, 15 days-PGJ2 has been demonstrated to inhibit macrophage activation and aggregation *in vivo* and *in vitro*, which might play a pivotal role in liver IRI (Han et al., 2012; Liu et al., 2012). In another study, treatment of mice with 15 days-PGJ2 reduced the serum expression levels of TNF-α, IL-1β, and F4/80 (a major biomarker of KCs) compared to the control group (Chen Z. et al., 2017). This may be due to the inhibitory effect on KC activation resulting in a comparatively moderate inflammatory response, including reduction in the elevated serum ALT and AST levels. Further examination indicated that hepatocyte necrosis and apoptosis were inhibited, thereby alleviating tissue damage. Autophagy was also inhibited by the activation of nuclear factor–erythroid related factor 2 (Nrf2), thereby enhancing the clearance of ROS and consequently suppressing HIF1α/BNIP3 and LC3 expression. However, their results were surprising, since hepatic PPARγ activation may only have weak inhibitory effects on the production of pro-inflammatory cytokines induced by 15 days-PGJ2. Based on this, it protected liver tissue against IRI, which was probably only partially dependent on PPARγ. This conclusion is consistent with that of a previous study by Kuboki et al., wherein they established a PPARγ^+/−^ mouse model with IR and demonstrated that activation of PPARγ was reduced in mice, and interestingly, after 8 h of reperfusion, they showed worse liver function than their wild-type counterparts. In other words, PPARγ^+/–^mice suffered more severe IRI (Kuboki et al., 2008). The partial PPARγ-dependent protective effects of 15 days-PGJ2 on IRI have also been observed in renal tissues. However, further research is required to identify the specific protective mechanism of PPARγ against liver IRI.

**TABLE 2:** Summarization of the effects of drugs acting on PPARγ in hepatic IRI.

Drugs act on PPARγ	IRI models	Main effects	Conclusion	Reference
15 daysPGJ2^a^	Balb/c mice (7 weeks, 22 ± 2 g)	Decreased serum TNF-α, IL-1β, F4/80, beclin-1, LC3, apoptotic cells, and autophagosomes. Upregulated Bcl-2/Bax ratio	15 days-PGJ2 protects liver IR injury *via* reducing Kupffer cell activation and activating the Nrf2 pathway	Chen K. et al. (2017)
15 days-PGJ2^a^	Balb/c mice (7 weeks, 22 ± 2 g)	Reduced serum TNF-a, IL-1b and ROS, inhibited apoptosis, and autophagic cell death	15 days-PGJ2 alleviates liver injury by up-regulating HO-1 and inhibiting hepatic cell autophagy	Chen et al. (2016)
Dexmedetomidine^b^	C57BL/6 mice (8 weeks)	Inhibited intrahepatic pro-inflammatory innate immune activation	Dexmedetomidine attenuates liver IRI *via* PPARγ/STAT3 pathway	Zhou et al. (2020)
Losartan^b^	C57BL/6 mice (8–10 weeks)	Reduced ALT activity, TNF-α and IL-6 levels, decreased in apoptosis	Losartan ameliorates liver IRI with PPARγ involvement, and inhibits RAGE-mediated signaling pathway	Koh et al. (2013)
Pioglitazone^c^	Wistar rats (200–250 g)	Reduced TNF-α, MDA, NADPH oxidase mRNA, apoptotic cell death, and oxidative stress, and increased Nrf2, PPARγ1, Hmox1, and TRx expression	PPARγ is a potential target to protect liver in patients with renal IRI	Elshazly and Soliman. (2019)
Rosiglitazone^c^	C57BL/6 (10–12 weeks)	Reduced apoptosis, necrosis, nitric oxide + Kupffer cell population, and increased CD206 + Kupffer cell population	PPARγ can be an essential tool to ameliorate liver outcomes by reducing the pro-inflammatory phenotype of KCs and IRI	Linares et al. (2018)

Nrf2, nuclear factor erythroid-related factor 2; RAGE, receptor for advanced glycation end product; MDA, malondialdehyde; Hmox1, hepatic hemoxygenase-1; TRx, hepatic thioredoxin.

a PPARγ natural ligand.

b PPARγ agonist.

c PPARγ synthetic ligand.

Interestingly, remote organ damage due to IR is also the major cause of death in patients, and studies have shown that PPARγ plays a pivotal role in this kind of disease in animal models. Pioglitazone rescues liver damage induced by renal IRI, and in addition, the decline in renal function due to IRI was also improved in rats. Mangiferin has the ability to protect against liver injury caused due to gut IR. In addition, the involvement of downstream cascade of NF-κB p65 phosphorylation, and the potential activation of PPARγ and 3β/β-catenin signaling pathways should be noted. Both the protective effects in organ IR-induced liver damage described above may partly be attributed to the antioxidant, anti-inflammatory, and anti-apoptotic effects of PPARγ (El-Sayyad et al., 2017; Elshazly and Soliman, 2019). Notably, the first member of the sequence similarity 3 (FAM3) gene family, FAM3A, has been identified as a novel target gene of PPARγ. Studies have shown that it has a protective effect against IRI. Previous studies have demonstrated that in mouse models of liver IR, knockdown of hepatic FAM3A significantly increased the risk of IRI. In addition, ATP production, Akt activity, and anti-apoptotic gene expression were all inhibited in the liver upon experimental examination. However, pretreatment with RGZ notably protected hepatic cells against IRI with the upregulation of FAM3A together with increasing ATP production and Akt activity (Zhou et al., 2013; Chen K. et al., 2017). To further investigate its explicit role, FAM3A-deficient mice were used for experimentation. They found that FAM3A-deficient mice exhibited more severe liver damage than wild-type (Wt) mice, and hepatic ATP production and Akt activity were markedly decreased in these mice. Moreover, RGZ pretreatment failed to reverse these outcomes. Therefore, they concluded that the molecular mechanism by which RGZ pretreatment ameliorated liver damage in Wt mice may be *via* the FAM3A-ATP-Akt pathway. Regarding the inflammatory response and oxidative stress, FAM3A has been shown to suppress NF-κB activity; hence, inflammation in mice hepatic cells induced by IR was decreased, due to reduced pro-inflammatory cytokine expression and inflammatory factor production. After RGZ treatment, NF-κB activity and oxidative stress were both significantly repressed in Wt mice livers, but aggravated liver damage in FAM3A-deficient mice, indicating that the hepatoprotective effects of FAM3A were associated with the activation of the ATP-PI3K-Akt pathway and suppression of NF-κB signaling, and hence alleviating oxidative stress in hepatocytes. A recent study on the drug that is widely used for sedation of patients undergoing surgery with anesthesia, dexmedetomidine, has been reported to mitigate liver damage after IR by facilitating macrophage M2 activation in a PPARγ/STAT3-dependent manner. Therefore, this might be a possible strategy to reduce the damage caused to the liver by IR during the perioperative period before the occurrence of liver surgery-induced IRI (Zhou et al., 2020).

## The Roles of Peroxisome Proliferator-Activated Receptor *γ* in Cerebral Ischemia-Reperfusion Injury

To our knowledge, brain tissues and spinal cord are more vulnerable to ischemic and hypoxic damage due to various factors than other tissues such as the kidney or liver. Neurons are sensitive to ischemia and hypoxia, and blocking the blood flow to the brain for only 5 min may result in neurocellular death. Inflammatory response, oxidative stress, blood–brain barrier (BBB) destruction, mitochondria-mediated mechanisms, and leukocyte infiltration are regarded as the various mechanisms involved in the pathophysiology of IRI (Posada-Duque et al., 2014; Lin et al., 2016). In this section, we review the role of PPARγ in cerebral IRI.

Similar to the protective roles in renal and hepatic IRI, pretreatment of rat or human neurons with 15 days-PGJ2 prevented H_2_O_2_-induced cytotoxicity and neuronal apoptosis. Upon intraventricular application, 15 days-PGJ2 has exhibited caspase 3 activity inhibiting effects in the ischemic cortex, thereby reducing cell apoptosis. In the ischemic cortex and cultured neurons, this phenomenon is mainly attributed to increased HO-1 expression, as well as suppressed COX-2 expression and NF-κB activation (Lin et al., 2006). The thiazolidinedione PPARγ agonist pioglitazone inhibits Ras-related C3 botulinum toxin substrate l (Rac1) activity, which has been identified as a small GTPase protein that is an essential subunit of NADPH oxidase for the generation of ROS. Moreover, Rac1 activation leads to a series of oxidative stress events in normal or diabetic brains, while inhibiting its activity and providing notable neuroprotective effects. By inhibiting the ischemia-induced cytoplasmic translocation of high-mobility, group protein 1 (HMGB-1) and the expression of advanced glycation end products (RAGE), pioglitazone triggered neuroinflammation, and microglial activation with the activation of the MAPK and NF-κB signaling pathways. In the PPARγ irreversible antagonist GW9662 treatment group, the neuroprotective effects in the *in vitro* IR model were abrogated, and knockdown of Rac1 showed the opposite neural outcomes of pioglitazone treatment compared to the control group. This suggested that IR-induced cerebral apoptosis was attenuated by pioglitazone *via* PPARγ and was accompanied by the suppression of HMGB-1/RAGE and Rac1/ROS activation (Zhang et al., 2009; Johanna et al., 2010; Raz et al., 2010; Xia et al., 2018). Additionally, these potential biological reactions account for BBB destruction, and accumulating evidence suggests involvement of inflammation, oxidative stress, toxic damage of excitotoxic amino acids, and intracellular calcium overload to be the essential pathological mechanisms of cerebral IRI (Rosenberg, 2012; Wu et al., 2015). It has been revealed that post-intracerebral hemorrhage and the administration of rosiglitazone could improve the BBB damage in rabbits. Therefore, illustrating its potential anti-inflammatory mechanism is not only beneficial for identifying methods to maintain BBB stability but also helpful to guide clinicians in the treatment of cerebral IRI. This is an interesting study topic for many neurobiology researchers. In the mouse middle cerebral artery occlusion and reperfusion (MCAO/R) model, following IRI, increased levels of serum ICAM-1, VCAM-1, P-selectin, L-selectin, and CD11b/CD18 were markedly improved by D-allose infusion. Additionally, D-allose suppressed the activation of NF-κB, a typical signaling pathway that regulates inflammatory response, in the MCAO/R model, in which the NF-κB-associated inflammatory effects were influenced severely. By administering GW9662, PPARγ expression was inhibited, and the expression of TNF-α and NF-κB p65 was enhanced. However, the GW9662 preconditioned cerebral IR outcomes were reversed by D-allose treatment. Taken together, it may exert an anti-inflammatory function *via* a PPARγ-mediated pathway, and BBB stability is a pivotal factor in preventing the progression of IRI (Huang et al., 2016). Similarly, 1,25-D3 also exhibits a protective anti-inflammatory role upon MCAO/R insult by maintaining BBB integrity through the activation of the PPARγ pathway accompanied by the upregulation of brain-derived neurotrophic factor (BDNF) owing to PPARγ activation (Guo et al., 2018).

Autophagy is a highly regulated process characterized by the formation of autophagosomes. When autophagosomes fuse with the lysosomes, autophagolysosomes are formed. Degenerated and damaged organelles as well as cytoplasmic macromolecules in the cells can be degraded by the autophagy–lysosomal system, which plays an important role in maintaining cell homeostasis. The role of autophagy in cerebral IRI is very complicated and has been reported to be related to brain maturity, brain area, severity of injury, and ischemic stage (Xu et al., 2012). Paradoxically, the role of autophagy in IR-induced neuronal death is controversial, and whether it is beneficial or harmful needs further research. The protective role of autophagy against IRI has been previously demonstrated (Liang et al., 1999). Further, extensive research has been conducted to examine the role of autophagy in organ IRI. It has been demonstrated that the PPARγ agonist 15 days-PGJ2 exerted neuroprotection against cerebral I/R injury by inhibiting neuronal autophagy (Xu et al., 2013). Another study has reported that the neuroprotection provided by 15 days-PGJ2 may be partially through inhibition of neuronal autophagy after oxygen–glucose deprivation/reoxygenation injury (Qin et al., 2015). The neuroprotective effect exerted by rosiglitazone may or may not rely on inhibiting the process of neuroinflammation and autophagic neuronal death, or may partially affect these processes in IR-induced cerebral dysfunction. A short period of ischemia followed by reperfusion applied to the organ may activate endogenous defense mechanisms that protect against a subsequent, sustained ischemic insult, a phenomenon known as ischemic preconditioning (IPC) (Bonventre, 2002). Interestingly, in focal ischemic preconditioning (IPC) and/or permanent focal cerebral ischemia (PFI) models that are induced by suture occlusion technique, administration of 3-MA before the onset of IPC showed that it attenuated the neuroprotective effects of ischemic preconditioning (Sheng et al., 2010). Moreover, rapamycin could mimic the neuroprotective effects of ischemic preconditioning. They proved that in the process of autophagy activation, cerebral IPC has significant tolerance to the concomitant fatal ischemia, and autophagy activators could stimulate the protective effects of IPC in cerebral ischemia. The different experimental results described above may indicate that autophagy plays a different role during the diverse stages of cerebral ischemia. During myocardial ischemia, autophagy may be beneficial in attenuating cell death, while on the contrary, detrimental aspects during reperfusion process have also been reported. The expression of autophagy-related molecular markers was strongly activated during reperfusion after cerebral ischemia, which indicates the possibility of greater cell damage after reperfusion. Moreover, the adverse effects of reperfusion were improved after inhibition of autophagy, further clarifying a detrimental role of autophagy during reperfusion (Matsui et al., 2007; Puyal and Clarke, 2009). The different effects of autophagy in IRI of different organs indicate the multifunctional and complicated role of autophagy in cerebral ischemia. Whether autophagy promotes cell survival or exaggerates the infarct volume may depend on the pathological situation. A better understanding of autophagy may provide new therapeutic targets for treating cerebral IRI. In addition, it is required that in studying the role and molecular mechanism of autophagy during IRI, the multilateral effects of autophagy should be taken into consideration. More information please see Table 3.

**TABLE 3:** Summarization of the effects of drugs acting on PPARγ in cerebral IRI.

Drugs act on PPARγ	IRI models	Main effects	Conclusion	Reference
15 daysPGJ2^a^	Male Long-Evans rats	Suppressed apoptosis and necrosis, and increased PPARγ, HO-1 expression	15 days-PGJ2 improves cerebral IRI insult, and decreases apoptosis and necrosis *via* a PPARγ-dependent way	Lin et al. (2006)
15 days-PGJ2^a^	Neuronal cells derived from the neocortices of E15 embryos in pregnant female C57BL/6 J mice	Protected neurons against cell death and inhibited neuronal autophagy	15 days-PGJ2 protects neurons partially by inhibiting autophagy *via* up-regulating Bcl-2 and inhibiting beclin1–Bcl2 heterodimer dissociation	Qin et al. (2015)
12-HETE^b^	Adult male Sprague–Dawley rats weighing 280–330 g	Suppressed iNOS expression, protected cortical neurons, and activated PPARγ	12-HETE exerts neuroprotective effect through PPARγ activation *via* the 12/15-lox pathway	Han et al. (2015)
Bexarotene^b^	Adult male Sprague–Dawley rats (280–320 g)	Improved neurobehavioral deficits and reduced brain edema, effects of microglia/macrophage activation and neutrophil infiltration	Bexarotene protects the brain at least in part through PPARγ/SIRT6/FoxO_3_, a signaling pathway	Zuo et al. (2019)
d-allose^b^	Male BALB/c mice weighing 20–25 g	Inhibited apoptosis, various inflammatory cytokines, and inflammation-related molecules	d-allose has therapeutic implication that may involve in the PPARγ-dependent activation	Huang et al. (2016)
Aleglitazar^b^	Fetal C57BL/6 N mice (E15) cerebral cortex	Anti-inflammation and reduction in NO production, release of pro-inflammatory cytokines, migration, and phagocytosis	Aleglitazar can be a clinical stroke therapy with short-term treatment	Boujon et al. (2019)
Icariin (ICA)^b^	Sprague–Dawley rats (4 months, 250–280 g)	Decreased neurological deficit score, diminished the infarct volume, and reduced the levels of IL-1β and TGF-β1	ICA has neuroprotective effects by inhibiting NF-κB, PPARα, and PPARγ mediate inflammation	Xiong et al. (2016)
Mifepristone^b^	Male Sprague–Dawley rats weighing 250–280 g	Reduced the levels of TNF-α, IL-1β, IL-6, MMP-2, and MMP-9, and increased TIMP-1 protein	Mifepristone relieves cerebral IRI by restoring the balance between MMPs and TIMPs and inhibiting inflammatory cytokines by activating PPARγ	Wu et al. (2018)
Rosiglitazone^c^	Female Sprague–Dawley rats weighing 250–300 g, aged 3 months	Reduced cerebral infarct volume, brain edema, attenuated IL-1β, IL-6, TNF-α, LC3-II/LC3-I and Beclin-1 level	Rosiglitazone may protect brain through inhibiting neuroinflammation and autophagic neuronal death	Shao and Liu (2015)
Umbelliferone^b^	Male Sprague–Dawley rats weight 220–270 g	Reduced MDA, IL1β, and IL-18; increased SOD, PPARγ level; suppressed the expression of NLRP3 inflammasome and TXNIP induction	Umbelliferone ameliorates cerebral IRI, may be partly related with the inhibition of NLRP3 inflammasome in the brain, and upregulation of PPARγ expression	Wang et al. (2015)

HO-1, heme oxygenase-1; E15, embryonic day 15; iNOS, inducible NO synthase; 12-HETE, 12-hydroxyeicosatetraenoic acid; SIRT6P, the endogenous retinoid X receptor and sirtuin 6; FoxO3a, forkhead box O3a; BBB, blood–brain barrier; MMP-2, , tissue inhibitor of metalloproteinase 1; TXNIP, thioredoxin interacting protein; MDA, malondialdehyde; NLRP3, nod-like receptor family, pyrin domain containing 3.

a PPARγ natural ligand.

b PPARγ agonist.

c PPARγ synthetic ligand.

## The Role of Peroxisome Proliferator-Activated Receptor *γ* in Myocardial Ischemia-Reperfusion Injury

Acute myocardial IRI (MIRI) is one of the clinically important causes of death in patients. It can induce irreversible pathological reactions in tissues, thus aggravating tissue damage. It can be divided into three types: myocardial stunning, reperfusion arrhythmia, and myocardial necrosis, manifested by myocardial cell necrosis, apoptosis, mitochondrial dysfunction, increased lipid peroxides, massive generation of free radicals, occurrence of malignant arrhythmia, weakened left ventricular contractility, and decreased intraventricular pressure (Baines, 2011; Kloner, 2013). During the pathophysiological process of IRI, it has been observed that tissues exhibit a certain antagonistic effect on IRI insult facilitated by PPARγ activation. The role of PPARγ in the heart remains to be fully elucidated. According to the literature, PPARγ is undoubtedly very important because of its remarkable insulin sensitivity and benefits. In this section, we review the role of PPARγ in MIRI.

As a class of highly active oxygen metabolites that interacts with antioxidants and causes oxidative stress (OS), nitric oxide (NO) is a contributing factor in the pathogenesis of many diseases, including myocardial IRI. Traditional synthetic PPARγ ligand pioglitazone could reduce myocardial infarct size and improve left ventricle (LV) function. Upon pretreatment with GW9662, wortmannin (PI3-kinase inhibitor), and nitric oxide synthase (NOS) inhibitor L-NAME, the reduced infarct size and improved LV function, due to pioglitazone, were abrogated by activated PPARγ, PI3-kinase, Akt, and endothelial NOS (eNOS) pathway in rabbits (Yasuda et al., 2009). In eNOS and inducible NOS (iNOS) knockout mice, myocardial infarct size was reduced in all the groups upon oral administration of pioglitazone. In contrast, in the eNOS and iNOS knockout mice groups, the myocardial protective effects of pioglitazone were obviously decreased compared to the wild-type group, accompanied by the upregulation and activation of cytosolic phospholipase (A2 (cPLA2), COX2, and PGI2 synthase; Ye et al., 2008). It has been reported that in cells exposed to IR, treatment with GW9662 alone or the COX2 inhibitor SC58125, had no effect on P-Akt levels, whereas wortmannin significantly decreased P-Akt levels (Birnbaum et al., 2011). PIO dramatically increased P-Akt levels. Wortmannin could block this effect, but GW9662 and SC58125 failed to alter the levels of these biomarkers. The cPLA2 level was slightly changed by GW9662, wortmannin, and SC58125 treatments, except for PIO. The increased cPLA2 level, due to PIO administration, was not alleviated by the effects of GW9662, wortmannin, and SC58125. Regarding COX2 levels, PIO induced a similar effect as cPLA2, and this outcome was blocked by SC58125 but not by GW9662 and wortmannin. In summary, the P-Akt levels in cardiomyocytes exposed to PIO were comparable in cells with intact or knocked-out PPARγ. PIO also significantly increased cPLA2 and COX-2 levels similarly in cells, regardless of the PPARγ status. This suggests that PIO upregulates the levels of Akt, cPLA2, and COX-2 in a PPARγ-independent manner because in cardiomyocytes, the levels of these enzymes are comparable with or without intact PPARγ. The difference in results from the above studies needs further investigation in order to verify whether the thiazolidinediones prevent myocardial IRI in animals in a PPARγ, cPLA2, and COX2 dependent manner. Another study found that upregulation of ERK and COX-2 induced by pioglitazone can abolish MIRI to a great extent (Wang et al., 2012). Pretreatment with quercetin suppressed H/R-induced NF-κB activation, thus attenuating the increased expression of iNOS, which protects against myocardial IRI by ameliorating OS. However, the inhibitory effect of the NF-κB pathway by quercetin was attenuated when PPARγ siRNA was used, which indicated that quercetin prevented NF-κB activation and reversed these antioxidative effects *via* PPARγ in cardiomyocytes with H/R (Liu et al., 2016). Notably, aleglitazar, a dual PPARα/γ agonist, significantly improves microvascular dysfunction and inhibits cell apoptosis, which is attributed to the stimulation of both PPARα/AKT/eNOS and PPARγ/AKT/eNOS signaling pathways (Qian et al., 2016).

Platelets are an important component of blood, and play an important role in coagulation, inflammation, host defense, and wound healing. Once activated, platelets can release inflammatory mediators that can either promote or inhibit tissue damage depending on the specific circumstance, growth factors, and proteases. Inhibition of platelet activity can reduce ischemic injury to the heart (Xu et al., 2006). One of the main causes of acute myocardial ischemia in humans is the rupture of atherosclerotic plaques, which induces circulating emboli formation, blocks blood vessels, and platelets accumulate on them, forming thrombi. Pioglitazone has been confirmed to decrease platelet aggregation and delay arterial thrombus formation in a mouse model, regardless of the dosage. Moreover, inhibition of platelet aggregation and delay of thrombosis may be associated with increased constitutive NOS (cNOS) and thrombomodulin expression in the endothelium of rat aorta (Li et al., 2005). Because of its ability to inhibit phosphodiesterase, cilostazol is currently used to inhibit platelet aggregation and dilate the arteries. In a mouse model of MIRI, PIO could significantly reverse the increase in pro-inflammatory factors (IL-1β, IL-6, and TNF-α) induced by MIRI, whereas cilostazol alone could not. Such changes were also observed in apoptotic proteins (Bax, caspase-3, and Bcl-2) when cilostazol was administered, and the PPARγ/JAK2/STAT3 pathway was involved (Li et al., 2017). The protective role of cilostazol in hippocampal neurons in brain IR models has also been reported. By means of PI3K-Akt1/JNK3/caspase-3–dependent mechanisms, it represents the advantages for neuron integrity, improved learning/memory ability as well as attenuated impairment of exercise/exploratory activities (Qi et al., 2016). Another study found that in patients with AMI undergoing coronary artery bypass grafting (CAGB), platelet PPARγ expression tended to decrease gradually at the reperfusion stage (Zhou et al., 2017). PPARγ deficiency dramatically increased electron transport chain complex (ETC) activity and mitochondrial oxidative phosphorylation *via* induction of FUN14 domain containing 1 (FUNDC1) dephosphorylation and subsequent mitophagy activation. This augmented the production of platelet ATP and eventually facilitated platelet aggregation and adhesion molecule expression, thereby accelerating the formation of micro-thromboses. Platelet overactivity is considered to be highly correlated with myocardial and microvascular hypoxia/reoxygenation damages, and melatonin can reverse PPARγ activation in mouse platelets under IR conditions, thereby strongly inhibiting mitochondrial autophagy required by FUNDC1. Upon melatonin treatment, abnormal mitochondrial autophagy of platelets leads to platelet accumulation and disorder, ultimately enhancing the duration of tissue IRI. In summary, melatonin treatment suppresses platelet activation and function against cardiac IRI, and the underlying mechanism may involve PPARγ/FUNDC1/mitophagy activation pathways.

**FIGURE 1:**
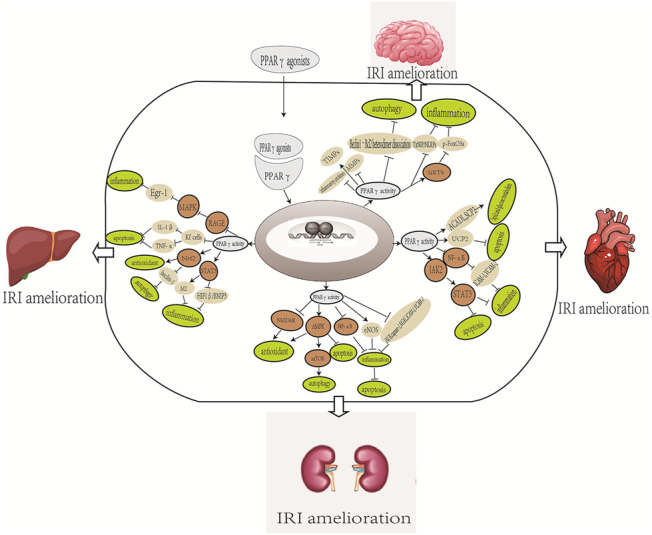
Overview of the possible mechanisms of PPARγ in the organ IRI. The mechanism includes different effects of different PPARγ agonists. NMDA, N-methyl-D-aspartic acid; eNOS, endothelial NO synthase; iNOS, inducible NO synthase; Nrf2, nuclear factor-erythroid 2-related factor 2; RAGE, receptor for advanced glycation end products; SIRT6P, the endogenous retinoid X receptor and sirtuin 6; Fox03a; BBB, blood–brain barrier; MMB, matrix metalloproteinase; TIMPs, tissue inhibitor of metalloproteinase; TXNIP, thioredoxin interacting protein; NLRP3, nod-like receptor family, pyrin domain containing 3; SCP2, sterol carrier protein 2; ACADI, long-chain acyl CoA dehydrogenase; UCP2, mitochondrial uncoupling protein 2.

The master transcription factor that is integral in inducing endogenous antioxidant enzymes in response to OS is the nuclear factor E2-associated factor 2 (Nrf2). It regulates cellular functions in myocardial IRI, including apoptosis, endoplasmic reticulum stress, mitochondrial function, inflammatory response, and autophagy. The ligand 15 days-PGJ2 plays a key role in augmenting the cellular antioxidant defense mechanism and reducing inflammation by suppressing NF-κB and activating PPARγ and Nrf2. Furthermore, coordinated activation of PPARγ and Nrf2 is a key signaling mechanism for chrysin to inhibit the negative impact on IR-induced OS, inflammation, and apoptosis. This finding was further validated by coadministering a PPARγ antagonist, GW9662, in which serum oxidative product, inflammatory cytokine, and apoptotic biomarker levels were significantly increased by inhibiting PPARγ/Nrf2 expression (Katsumata et al., 2014; Rani and Arya, 2020). More information about the role of PPARγ in myocardial IRI is summarized in Table 4 and the possible mechanisms of PPARγ in Figure 1.

**TABLE 4:** Summarization of the effects of drugs acting on PPARγ in myocardium IRI.

Drugs act on PPARγ	IRI models	Main effects	Conclusion	Refeference
Cilostazol^a^	Male C57BL6/J mice (35 ± 5 g)	Reduced IL-1b, IL-6, TNF-α, Bax, caspase-3, and Bcl-2	Cilostazol suppresses apoptosis and pro-inflammatory reactions *via* pparγ/jak2/stat3 pathway	Li et al. (2017)
Telmisartan^a^	Male Wistar albino rats (8–9 weeks, 200–250 g)	Ameliorated activities of antioxidants, CK-MB, LDH, TNF-α, MDA, and Bax expression	Telmisartan has a beneficial effect in IRI that may be partially dependent on PPARγ	Goyal et al. (2011)
Rosuvastatin^a^	New Zealand white rabbits (4.0–5.0 kg, 6 months)	Downregulated caspase-9 and cyt c expression, and upregulated UCP2 and PPARγ expression	Rosuvastatin mitigates myocardial IRI by up-regulating PPARγ and UCP2	Wang et al. (2018)
Danqi pill^a^	SD rats in specific pathogen-free (SPF) grade	Improved myocardial function. Upregulated ACADL and SCP2	Danqi pill protects against myocardial IRI through PPARγ-mediated lipid and glucose metabolism regulation	Zhang et al. (2018)
β-Sitosterol^a^	The rat cardiomyocyte cell line (H9c2)	Reduced cell apoptosis, caspase-3 and 9, NF-κB protein levels, and increased in Bcl-2, PPARγ protein level	β-sitosterol may involve in the modulation of pparγ/NF-κB signaling during myocardial IRI	Lin et al. (2020)
Rosiglitazone^b^	Male FVB/NJ mice	Decreased myocardial infarction and improved postischemic recovery	Rosiglitazone can reduce heart ischemic injury *via* regulation of AMPK, akt, and JNK signaling pathways	Morrison et al. (2011)
Aleglitazar^a^	Cardiomyocytes from PPARγ knockout or wild-type mice; male db/db mice and their wild-type nondiabetic	Increased cell viability, P-Akt/P-eNOS level, and reduced apoptosis	The cardioprotective effects of aleglitazar are dependent on activation of both PPARα and PPARγ	Qian et al. (2016)
Pioglitazone^b^	Adult male C57BL/6 J mice (9–13 weeks old)	Reduced myocardial IRI; antagonized monocyte/macrophage-mediated inflammation and induced macrophage polarization	Pioglitazone prevents the heart from IRI and cardiac remodeling by antagonizing acute inflammation	Tokutome et al. (2019)
Simvastatin	Male Sprague–Dawley rats (450–550 g)	Decreased the tissue level of IL-6, TNF-α, and MCP-1	The anti-inflammatory effects of simvastatin may be partially dependent on the activation of PPARγ	Shen et al. (2010)

MDA, malondialdehyde; SCP2, sterol carrier protein 2; ACADL, long-chain acyl CoA dehydrogenase; UCP2, mitochondrial uncoupling protein 2; HCMs, human cardiomyocytes; AMI, acute myocardial infarction; MCP-1, monocyte chemoattractant protein-1.

a PPARγ agonist.

b PPARγ synthetic ligand.

c PPARγ natural ligand.

## Peroxisome Proliferator-Activated Receptor *γ* in the Other Tissues of Ischemia-Reperfusion Injury

IRI not only emerges in the organs mentioned above but also rarely reports about PPARγ in other types of IRI. Here, we review the information on PPARγ in other types of IRI, their biological characteristics, and therapeutic perspectives.

Cilostazol could suppress apoptosis and pro-inflammatory reactions *via* the PPARγ/JAK2/STAT3 pathway in myocardium IRI (Li et al., 2017). A new mechanistic study by Gendy et al. (Gendy et al., 2021) disclosed that it could protect the mesenteric IR-induced lung lesion by intensifying the PPARγ expression and accompanied with the reducing content of NF-қB-p65 and STAT3. Thereby, the antioxidant capacity as well as the anti-inflammatory effect was intensifying; apoptotic cells were confirmed to be decreasing by enhancing Bcl-2 content and lessening caspase-3 level eventually.

Ovarian torsion is a dangerous gynecological emergency condition. Complete or partial rotation of the adnexa may induce ovarian IRI. By administering PIO orally before ovarian IRI was induced in rats, the inflammatory, apoptosis and oxidant stress were all alleviated by the biomarker detection. In addition, the well-known PIO target HO-1 level was also increased (Refaie and El-Hussieny, 2018). The anti-inflammatory response of PPARγ was also certified in retinal ganglion cells IRI *via* inhibiting NF-κB and p38 phosphorylation and the following TLR4/NLRP3 inflammasome activation (Zhang et al., 2013; Zhang et al., 2017).

What is more, protective effects of activating the PPARγ/NF-κB pathway against IRI were common in intestinal IRI (Nakajima et al., 2001; Liu et al., 2020). The involvement of the PPARγ/NF-κB signaling pathway, together with the Nrf2/HO-1 signaling pathways, by intervention of mangiferin was demonstrated to mitigate gastric ulcer, may intimate protective mechanisms partially, by modulation of oxidative stress, inflammation, and apoptosis possibly (Mahmoud-Awny et al., 2015).

## The Adverse Roles of Peroxisome Proliferator-Activated Receptor γ

Notably, despite the diverse functions of PPARγ in organ protection, its adverse role deserves attention. Increased subcutaneous adipose tissue or decreased visceral fat content may lead to weight gain upon TZD treatment. Fluid retention with associated edema is another serious adverse effect of TZDs, which is caused by increased sodium and water reabsorption in the kidney. It may also contribute to congestive heart failure. However, knockout of PPARγ in the collecting ducts has been shown to suppress the increase in plasma volume and body weight caused by TZDs (Guan et al., 2005; Zhang et al., 2005). Animal model studies have shown that TZDs cause bone loss by simultaneously enhancing osteoclastogenesis and inhibiting osteoblastogenesis, resulting in higher rate of fractures with lower bone mineral density (Wei and Wan, 2011). Moreover, PPARγ agonists have also been associated with an increased incidence of subcutaneous sarcomas and elevated risk of bladder cancer, and tumor promotion may rely on PPARγ activation (Azoulay et al., 2012; Pruimboom-Brees et al., 2012). It has been reported that PPARγ agonists can promote tumor development by activating PPARγ, although they play no role in tumor initiation (Vitale et al., 2016).

Paradoxically, PPARγ ligands have been reported to be unsuccessful in attenuating organ IRI in some studies. It was shown that RSG therapy had no obvious effect on reducing the IR myocardial infarct size in pigs. This was speculated to be attributable to moderate degree of myocardial ischemia, suggesting that the cardioprotective effects of RSG may be beneficial only during higher degree of ischemia (Xu et al., 2005). They reported that acute preperfusion with a high dose of troglitazone did not attenuate myocardial IRI in pigs. However, the susceptibility of cardiomyocytes to ventricular fibrillation was increased, which was associated with ion channel dysfunction, possibly *via* a PPARγ-independent mechanism (Xu et al., 2003). This finding was further confirmed by Riess et al. They performed dose–response experiments in hearts using rosiglitazone and found a remarkable increase in mitochondrial oxidation induced by rosiglitazone, as observed by reduced NADH and increased FAD autofluorescence (Riess et al., 2020). Failure of GW9662 to abolish these events indicated that increase in mitochondrial oxidation occurred in a PPARγ-independent manner. *In vivo* and *in vitro* studies in mice (He et al., 2014) reported that rosiglitazone boosts OS activity and mitochondrial dysfunction after IR in a PPARγ-independent manner by inhibiting SOD complexes I and IV, leading to mitochondrial dysfunction. Neither the inherited PPARγ deletion nor administration of PPARγ antagonist could reverse these adverse effects. Moreover, during rosiglitazone administration, decreased ATP production and deterioration of cardiac function were observed parallel to the above findings. Recently, a study using heart tissue isolated from rat suggested a dose-dependent and reversible mitochondrial oxidation by two different TZDs independent of PPARγ activation. Owing to the excessive oxidation of mitochondria, the production of intracellular ATP was decreased and the production of ROS was increased. These cellular events can damage the myocardium or increase their sensitivity to unexpected IRI. These findings may help explain the reported increase in adverse cardiac events (Riess et al., 2020; Table 5). Together, these studies reveal that PPARγ acts on various types of tissues to confer cellular metabolism and cause deleterious side effects. It seems that these side effects of PPARγ agonists are not all PPARγ dependent. The development of tissue-specific compounds that improve the differential between beneficial and adverse events is of special significance to translate the PPARγ biology into the clinic.

**TABLE 5:** Main PPARγagonists and their therapeutic benefits vs. drawbacks.

PPARγagonists	Main benefits	Main drawbacks	Reference
Pioglitizone	Increases insulin sensitivity and antidiabetic activity	Edema, weight gain, increased subcutaneous sarcomas, and bladder cancer risks	Aronoff et al. (2000), Raskin et al. (2000)
			Guan et al. (2005), Zhang et al. (2005), Azoulay et al. (2012), Pruimboom-Brees et al. (2012)
Rosiglitazone	Increases insulin sensitivity and antidiabetic activity. Improves blood pressure. Attenuates systemic inflammation	Edema, weight gain, increase in cardiovascular incidence, and osteoporosis risk	Rogue et al. (2010), Piche et al. (2018)
			Guan et al. (2005), Zhang et al. (2005), Nissen and Wolski. (2007)
Troglitazone	Increases insulin sensitivity and antidiabetic activity. Attenuates systemic inflammation	Severe liver injury and acute liver failure. Tumor promoting and pro-angiogenic properties	Rogue et al. (2010)
			Caballero et al. (2003), Chojkier (2005)
RS5444(Inolitazone or efatutazone)	Anticancer activity	Not specified	Shimazaki et al. (2008)
Farglitazar	Lipid-altering and antidiabetic activity	Significant edema	Willson et al. (2000)
			Henke et al. (1998)
S26948	Potent antidiabetic and antiatherogenic effects	Not specified	Sohn et al. (2009)
INT131	Lower plasma glucose without typical thiazolidinedione side effects	Not specified	Dunn et al. (2011)

It is worth noting that the hypoglycemic drug aleglitazar is a joint agonist with dual PPARα and PPARγ activation activity (Qian et al., 2016). The familiar PPARα hypolipidemic drug bezafibrate can stimulate three PPAR subtypes, which may have the potential to directly improve insulin sensitivity through PPARγ activation (Berger and Wagner, 2002). PPARα and *γ* stimulation play complementary roles in preventing atherosclerosis. Therefore, for diabetic patients with dyslipidemia, drugs with dual PPARα/PPARγ activity seem to be suitable for treatment. Activation of PPARα could reflect a compensatory response to the metabolic-shifted, apoptotic, and hypertrophic status of the hypertensive-diabetic cardiomyopathy (Ares-Carrasco et al., 2012). Fibrates have shown more selectivity and high potency as PPARα agonists, and they have been used in the treatment of atherogenic dyslipidemia and hypercholesterolemia (Nissen et al., 2007). PPARβ/δ was shown to be involved in lipid catabolism, glucose homeostasis, inflammation, survival, proliferation, differentiation, as well as mammalian regeneration of the skin, bone, and liver (Magadum and Engel, 2018). Evidences showed the effects of pioglitazone and fenofibrate in the expression of genes responsible for insulin resistance, fatty acid synthesis, and fibrosis, and in adiponectin. The activators of PPARα and PPARγ receptors can simultaneously reduce atherogenic triglycerides, raise cardioprotective HDL levels, and improve insulin resistance (Plutzky, 2012). Hence, dual activation of PPARγ and PPARα may be beneficial for cardiovascular disease risk reduction. Both the interaction and independent in metabolic regulation means considerable efforts are needed with a goal to design, synthesize, and characterize new highly potent and efficacious single, dual, and pan-PPARβ/δ or PPARγ agonists. The cross talk between clinical PPAR agonists suggests that it is necessary to conduct in-depth research on these drugs, including their respective, potential new therapeutic, as well as side effects, thereby providing theoretical guidance for clinical treatment. At the same time, their role in the pattern of multiple PPAR activations during IRI should also be considered.

## Conclusion

Considering their diverse actions on cell proliferation, apoptosis, and autophagy, PPARγ and its modulators need to undergo multiple experimental and clinical evaluations before their formal application as prospective IRI therapeutics. Natural ligands and their close derivatives are promising candidates as target drugs against IR diseases in the future. Even though the traditional PPARγ agonists have many unwanted side effects, there are no substitute values for treatment in cancer or IRI warrant identification. Once manifested, PPARγ, especially dual or pan-PPAR agonists, could be beneficial.

## Summary and Perspective

Recently, PPARγ and its agonists have attracted much attention as a promising developed medical technology capable of treating various organ IRI. Therefore, they have been extensively studied as potential therapies for IRI. Through our review and previous studies, we believe that the protective molecular mechanisms of PPARγ in different types of IRI may be interacted mutually. They can affect apoptosis, autophagy, inflammation, oxidation, *etc*. By bioinformatic analysis and a dual-luciferase reporter assay, PPARγ was identified as a target gene of miR-27a (Chi et al., 2019). For the molecular mechanism of PPARγ signaling, microRNA, or other noncoding RNA (circular RNA, long noncoding RNA, *etc*.), their functions between PPARγ signaling and IRI merit further discussion, if possible. We speculate that the discriminate molecular mechanism of PPARγ’s protective effects on IRI in different organs may be due to the specificity of tissues and insufficient research, thus lots of researches are needed before the role of PPARγ in IRI can be elucidated.

## References

[B1] Abdelrahman M. Collin M. Thiemermann C. (2004). The peroxisome proliferator-activated RECEPTOR-γ ligand 15-DEOXYD12,14 prostaglandin J2 reduces the organ injury IN hemorrhagic SHOCK. Shock 22, 555–561. 10.1097/01.shk.0000144132.13900.24 15545828

[B2] Akahori T. Sho M. Hamada K. Suzaki Y. Kuzumoto Y. Nomi T. (2007). Importance of peroxisome proliferator-activated receptor-γ in hepatic ischemia/reperfusion injury in mice. J. Hepatol. 47, 784–792. 10.1016/j.jhep.2007.07.030 17936399

[B3] Ares-Carrasco S. Picatoste B. Camafeita E. Carrasco-Navarro S. Zubiri I. Ortiz A. (2012). Proteome changes in the myocardium of experimental chronic diabetes and hypertension. J. Proteomics 75 (6), 1816–1829. 10.1016/j.jprot.2011.12.023 22234359

[B4] Aronoff S. Rosenblatt S. Braithwaite S. Egan J. W. Mathisen A. L. Schneider R. L. (2000). Pioglitazone hydrochloride monotherapy improves glycemic control in the treatment of patients with type 2 diabetes: a 6-month randomized placebo-controlled dose-response study. The Pioglitazone 001 Study Group. Diabetes Care 23, 1605–1611. 10.2337/diacare.23.11.1605 11092281

[B5] Azoulay L. Yin H. Filion K. B. Assayag J. Majdan A. Pollak M. N. (2012). The use of pioglitazone and the risk of bladder cancer in people with type 2 diabetes: nested case-control study. BMJ 344, e3645. 10.1136/bmj.e3645 22653981 PMC3365142

[B6] Baines C. P. (2011). How and when do myocytes die during ischemia and reperfusion: the late phase. J. Cardiovasc. Pharmacol. Ther. 16, 239–243. 10.1177/1074248411407769 21821522

[B7] Ballow A. Gader A. M. A. Huraib S. Al-Husaini K. Mutwalli A. Al-Wakeel J. (2005). Platelet surface receptor activation in patients with chronic renal failure on hemodialysis, peritoneal dialysis and those with successful kidney transplantation. Platelets 16, 19–24. 10.1080/09537100412331272569 15763892

[B8] Barish G. D. Narkar V. A. Evans R. M. (2006). PPAR delta: a dagger in the heart of the metabolic syndrome. J. Clin. Invest. 116, 590–597. 10.1172/JCI27955 16511591 PMC1386117

[B9] Ben-Ari Z. Pappo O. Yitzhaki S. Cheporko Y. Shainberg A. Zinman T. (2009). Uridine-5’-triphosphate protects against hepatic- ischemic/reperfusion injury in mice. Transplantation 87, 1155–1162. 10.1097/TP.0b013e31819e3cdc 19384161

[B10] Berger J. Wagner J. A. (2002). Physiological and therapeutic roles of peroxisome proliferator-activated receptors. Diabetes Tech. Ther. 4, 163–174. 10.1089/15209150260007381 12079620

[B11] Betz B. Schneider R. Kress T. Schick M. A. Wanner C. Sauvant C. (2012). Rosiglitazone affects nitric oxide synthases and improves renal outcome in a rat model of severe ischemia/reperfusion injury. PPAR Res. 2012, 219319. 10.1155/2012/219319 22448163 PMC3289925

[B12] Bhaumik P. Koski M. K. Glumoff T. Hiltunen J. K. Wierenga R. K. (2005). Structural biology of the thioester-dependent degradation and synthesis of fatty acids. Curr. Opin. Struct. Biol. 15 (6), 621–628. 10.1016/j.sbi.2005.10.010 16263264

[B13] Bianchi A. Moulin D. Hupont S. Koufany M. Netter P. Reboul P. (2014). Oxidative stress-induced expression of HSP70 contributes to the inhibitory effect of 15d-PGJ2 on inducible prostaglandin pathway in chondrocytes. Free Radic. Biol. Med. 76, 114–126. 10.1016/j.freeradbiomed.2014.07.028 25106704

[B14] Birnbaum Y. Long B. Qian J. Perez-Polo J. R. Ye Y. (2011). Pioglitazone limits myocardial infarct size, activates Akt, and upregulates cPLA2 and COX-2 in a PPAR-γ-independent manner. Basic Res. Cardiol. 106, 431–446. 10.1007/s00395-011-0162-3 21360043

[B15] Bonde M. M. Olsen K. B. Erikstrup N. Speerschneider T. Lyngsø C. Haunsø S. (2011). The angiotensin II type 1 receptor antagonist Losartan binds and activates bradykinin B2 receptor signaling. Regul. Peptides 167, 21–25. 10.1016/j.regpep.2010.11.003 21115072

[B16] Bonventre J. V. (2002). Kidney ischemic preconditioning. Curr. Opin. Nephrol. Hypertens. 11, 43–48. 10.1097/00041552-200201000-00007 11753086

[B17] Boujon V. Uhlemann R. Wegner S. Wright M. B. Laufs U. Endres M. (2019). Dual PPARα/γ agonist aleglitazar confers stroke protection in a model of mild focal brain ischemia in mice. J. Mol. Med. 97, 1127–1138. 10.1007/s00109-019-01801-0 31147725 PMC6647083

[B18] Bozic M. Valdivielso J. M. (2015). The potential of targeting NMDA receptors outside the CNS. Expert Opin. Ther. Targets 19 (3), 399–413. 10.1517/14728222.2014.983900 25495517

[B19] Caballero A. E. Saouaf R. Lim S. C. Hamdy O. Abou-Elenin K. O'Connor C. (2003). The effects of troglitazone, an insulin-sensitizing agent, on the endothelial function in early and late type 2 diabetes: a placebo-controlled randomized clinical trial. Metabolism 52, 173–180. 10.1053/meta.2003.50023 12601628

[B20] Cannistrà M. Ruggiero M. Zullo A. Gallelli G. Serafini S. Maria M. (2016). Hepatic ischemia reperfusion injury: a systematic review of literature and the role of current drugs and biomarkers. Int. J. Surg. 33 (Suppl. 1), S57–S70. 10.1016/j.ijsu.2016.05.050 27255130

[B21] Casillas-Ramirez A. Amine-Zaouali M. Massip-Salcedo M. Padrissa-Altés S. Bintanel-Morcillo M. Ramalho F. (2008). Inhibition of angiotensin II action protects rat steatotic livers against ischemia-reperfusion injury. Crit. Care Med. 36, 1256–1266. 10.1097/CCM.0b013e31816a023c 18379253

[B22] Chatterjee P. Patel N. S. Cuzzocrea S. Brown P. A. Stewart K. N. Mota-Filipe H. (2004). The cyclopentenone prostaglandin 15-deoxy-Δ12,14-prostaglandin J2 ameliorates ischemic acute renal failure. Cardiovasc. Res. 61, 630–643. 10.1016/j.cardiores.2003.10.024 14962493

[B23] Chen K. Li J. Li S. Feng J. Wu L. Liu T. (2016). 15D-PGJ2 alleviates ConA-induced acute liver injury in mice by up-regulating HO-1 and reducing hepatic cell autophagy. Biomed. Pharmacother. 80, 183–192. 10.1016/j.biopha.2016.03.012 27133055

[B24] Chen K. Li J.-J. Li S.-N. Feng J. Liu T. Wang F. (2017). 15-Deoxy-Δ12,14-prostaglandin J2 alleviates hepatic ischemia-reperfusion injury in mice via inducing antioxidant response and inhibiting apoptosis and autophagy. Acta Pharmacol. Sin. 38, 672–687. 10.1038/aps.2016.108 28216619 PMC5457695

[B25] Chen Z. Wang J. Yang W. Chen J. Meng Y. Geng B. (2017). FAM3A mediates PPARgamma's protection in liver ischemia-reperfusion injury by activating Akt survival pathway and repressing inflammation and oxidative stress. Oncotarget 8, 49882–49896. 10.18632/oncotarget.17805 28562339 PMC5564815

[B26] Chen W. Xi X. Zhang S. Zou C. Kuang R. Ye Z. (2018). Pioglitazone protects against renal Ischemia-Reperfusion injury via the AMP-Activated protein Kinase-Regulated autophagy pathway. Front. Pharmacol. 9, 851. 10.3389/fphar.2018.00851 30127742 PMC6088275

[B27] Chi X. Jiang Y. Chen Y. Yang F. Cai Q. Pan F. (2019). Suppression of microRNA-27a protects against liver ischemia/reperfusion injury by targeting PPARγ and inhibiting endoplasmic reticulum stress. Mol. Med. Rep. 20 (5), 4003–4012. 10.3892/mmr.2019.10645 31485635

[B28] Chinetti G. Fruchart J. C. Staels B. (2003). Peroxisome proliferator-activated receptors: new targets for the pharmacological modulation of macrophage gene expression and function. Curr. Opin. Lipidol. 14, 459–468. 10.1097/00041433-200310000-00006 14501584

[B29] Chojkier M. (2005). Troglitazone and liver injury: in search of answers. Hepatology 41, 237–246. 10.1002/hep.20567 15657914

[B30] Chouchani E. T. Pell V. R. Gaude E. Aksentijevic D. Sundier S. Y. Robb E. L. (2014). Ischaemic accumulation of succinate controls reperfusion injury through mitochondrial ROS. Nature 515, 431–435. 10.1038/nature13909 25383517 PMC4255242

[B31] Cuzzocrea S. (2004). Peroxisome proliferator-activated receptors gamma ligands and ischemia and reperfusion injury. Vascul Pharmacol. 41, 187–195. 10.1016/j.vph.2004.10.004 15653094

[B32] Decuypere J. P. Ceulemans L. J. Agostinis P. Monbaliu D. Naesens M. Pirenne J. (2015). Autophagy and the kidney: implications for ischemia-reperfusion injury and therapy. Am. J. Kidney Dis. 66 (4), 699–709. 10.1053/j.ajkd.2015.05.021 26169721

[B33] Docherty N. G. Lopez-Novoa J. M. Arevalo M. Duwel A. Rodriguez-Pena A. Pérez-Barriocanal F. (2006). Endoglin regulates renal ischaemia-reperfusion injury. Nephrol. Dial. Transpl. 21, 2106–2119. 10.1093/ndt/gfl179 16751653

[B34] Dunn F. L. Higgins L. S. Fredrickson J. DePaoli A. M. (2011). Selective modulation of PPARgamma activity can lower plasma glucose without typical thiazolidinedione side-effects in patients with Type 2 diabetes. J. Diabetes Complications 25, 151–158. 10.1016/j.jdiacomp.2010.06.006 20739195

[B35] El-Sayyad S. M. Soubh A. A. Awad A. S. El-Abhar H. S. (2017). Mangiferin protects against intestinal ischemia/reperfusion-induced liver injury: involvement of PPAR-gamma, GSK-3beta and Wnt/beta-catenin pathway. Eur. J. Pharmacol. 809, 80–86. 10.1016/j.ejphar.2017.05.021 28506911

[B36] Elshazly S. Soliman E. (2019). PPAR gamma agonist, pioglitazone, rescues liver damage induced by renal ischemia/reperfusion injury. Toxicol. Appl. Pharmacol. 362, 86–94. 10.1016/j.taap.2018.10.022 30393147

[B37] Evans R. M. Barish G. D. Wang Y. X. (2004). PPARs and the complex journey to obesity. Nat. Med. 10, 355–361. 10.1038/nm1025 15057233

[B38] Fajas L. Auboeuf D. Raspe E. Schoonjans K. Lefebvre A. M. Saladin R. (1997). The organization, promoter analysis, and expression of the human PPARgamma gene. J. Biol. Chem. 272, 18779–18789. 10.1074/jbc.272.30.18779 9228052

[B39] Fleisher T. A. (1997). Apoptosis. Ann. Allergy Asthma Immunol. 78 (3), 245–249. 10.1016/S1081-1206(10)63176-6 9087147

[B40] Friedewald J. J. Rabb H. (2004). Inflammatory cells in ischemic acute renal failure. Kidney Int. 66, 486–491. 10.1111/j.1523-1755.2004.761_3.x 15253694

[B41] Garcia I. Innis-Whitehouse W. Lopez A. Keniry M. Gilkerson R. (2018). Oxidative insults disrupt OPA1-mediated mitochondrial dynamics in cultured mammalian cells. Redox Rep. 23, 160–167. 10.1080/13510002.2018.1492766 29961397 PMC6272060

[B42] Gendy A. M. Amin M. M. Al-Mokaddem A. K. Abd Ellah M. F. (2021). Cilostazol mitigates mesenteric ischemia/reperfusion-induced lung lesion: contribution of PPAR-γ, NF-κB, and STAT3 crosstalk. Life Sci. 266, 118882. 10.1016/j.lfs.2020.118882 33310046

[B43] Glick D. Barth S. Macleod K. F. (2010). Autophagy: cellular and molecular mechanisms. J. Pathol. 221 (1), 3–12. 10.1002/path.2697 20225336 PMC2990190

[B44] Goyal S. N. Bharti S. Bhatia J. Nag T. C. Ray R. Arya D. S. (2011). Telmisartan, a dual ARB/partial PPAR-gamma agonist, protects myocardium from ischaemic reperfusion injury in experimental diabetes. Diabetes Obes. Metab. 13, 533–541. 10.1111/j.1463-1326.2011.01377.x 21320264

[B45] Guan Y. Hao C. Cha D. R. Rao R. Lu W. Kohan D. E. (2005). Thiazolidinediones expand body fluid volume through PPARgamma stimulation of ENaC-mediated renal salt absorption. Nat. Med. 11, 861–866. 10.1038/nm1278 16007095

[B46] Guo T. Wang Y. Guo Y. Wu S. Chen W. Liu N. (2018). 1, 25-D3 protects from cerebral ischemia by maintaining BBB permeability via PPAR-gamma activation. Front Cel Neurosci. 12, 480. 10.3389/fncel.2018.00480 PMC6304345 30618630

[B47] Gupta R. A. Wang D. Katkuri S. Wang H. Dey S. K. DuBois R. N. (2004). Activation of nuclear hormone receptor peroxisome proliferator-activated receptor-delta accelerates intestinal adenoma growth. Nat. Med. 10, 245–247. 10.1038/nm993 14758356

[B48] Han H. Shin S. W. Seo C. Y. Kwon H. C. Han J. Y. Kim I. H. (2007). 15-Deoxy-delta 12,14-prostaglandin J2 (15d-PGJ 2) sensitizes human leukemic HL-60 cells to tumor necrosis factor-related apoptosis-inducing ligand (TRAIL)-induced apoptosis through Akt downregulation. Apoptosis 12, 2101–2114. 10.1007/s10495-007-0124-2 17786557

[B49] Han J. Sun L. Xu Y. Liang H. Cheng Y. (2015). Activation of PPARgamma by 12/15-lipoxygenase during cerebral ischemia-reperfusion injury. Int. J. Mol. Med. 35, 195–201. 10.3892/ijmm.2014.1998 25395029

[B50] Han Z. Zhu T. Liu X. Li C. Yue S.(2012). 15-Deoxy-Delta12,14 -prostaglandin J2 reduces recruitment of bone marrow-derived monocyte/macrophages in chronic liver injury in mice. Hepatology 56, 350–360. 10.1002/hep.25672 22371273

[B51] Haraguchi G. Kosuge H. Maejima Y. Suzuki J. Imai T. Yoshida M. (2008). Pioglitazone reduces systematic inflammation and improves mortality in apolipoprotein E knockout mice with sepsis. Intensive Care Med. 34, 1304–1312. 10.1007/s00134-008-1024-9 18283431

[B52] He H. Tao H. Xiong H. Duan S. Z. McGowan F. J. Mortensen R. M. (2014). Rosiglitazone causes cardiotoxicity via peroxisome proliferator-activated receptor gamma-independent mitochondrial oxidative stress in mouse hearts. Toxicol. Sci. 138, 468–481. 10.1093/toxsci/kfu015 24449420 PMC4243497

[B53] Henke B. R. Blanchard S. G. Brackeen M. F. Brown K. K. Cobb J. E. Collins J. L. (1998). N-(2-Benzoylphenyl)-L-tyrosine PPARgamma agonists. 1. Discovery of a novel series of potent antihyperglycemic and antihyperlipidemic agents. J. Med. Chem. 41, 5020–5036. 10.1021/jm9804127 9836620

[B54] Hu H. Zou C. Xi X. Shi Z. Wang G. Huang X. (2012). Protective effects of pioglitazone on renal ischemia-reperfusion injury in mice. J. Surg. Res. 178, 460–465. 10.1016/j.jss.2012.01.012 22507688

[B55] Huang T. Gao D. Hei Y. Zhang X. Chen X. Fei Z. (2016). D-allose protects the blood brain barrier through PPARgamma-mediated anti-inflammatory pathway in the mice model of ischemia reperfusion injury. Brain Res. 1642, 478–486. 10.1016/j.brainres.2016.04.038 27103568

[B56] Inceoglu B. Bettaieb A. Haj F. G. Gomes A. V. Hammock B. D. (2017). Modulation of mitochondrial dysfunction and endoplasmic reticulum stress are key mechanisms for the wide-ranging actions of epoxy fatty acids and soluble epoxide hydrolase inhibitors. Prostaglandins Other Lipid Mediat. 133, 68–78. 10.1016/j.prostaglandins.2017.08.003 28847566 PMC5824649

[B57] Ji J. Wu L. Feng J. Mo W. Wu J. Yu Q. (2020). Cafestol preconditioning attenuates apoptosis and autophagy during hepatic ischemia-reperfusion injury by inhibiting ERK/PPARgamma pathway. Int. Immunopharmacol 84, 106529. 10.1016/j.intimp.2020.106529 32344356

[B58] Johanna G. V. Fredy C. A. David V. C. Natalia M. V. Angel C. R. Gloria Patricia C-G. (2010). Rac1 activity changes are associated with neuronal pathology and spatial memory long-term recovery after global cerebral ischemia. Neurochem. Int. 57, 762–773. 10.1016/j.neuint.2010.08.014 20817060

[B59] Kalogeris T. Baines C. P. Krenz M. Korthuis R. J. (2012). Cell biology of ischemia/reperfusion injury. Int. Rev. Cel Mol Biol 298, 229–317. 10.1016/B978-0-12-394309-5.00006-7 PMC3904795 22878108

[B60] Katsumata Y. Shinmura K. Sugiura Y. Tohyama S. Matsuhashi T. Ito H. (2014). Endogenous prostaglandin D2 and its metabolites protect the heart against ischemia-reperfusion injury by activating Nrf2. Hypertension 63, 80–87. 10.1161/HYPERTENSIONAHA.113.01639 24101662

[B61] Kieran N. E. Rabb H. (2004). Immune responses in kidney preservation and reperfusion injury. J. Investig. Med. 52, 310–314. 10.1136/jim-52-05-30 15551653

[B62] Kimura T. Takabatake Y. Takahashi A. Kaimori J. Y. Matsui I. Namba T. (2011). Autophagy protects the proximal tubule from degeneration and acute ischemic injury. J. Am. Soc. Nephrol. 22, 902–913. 10.1681/ASN.2010070705 21493778 PMC3083312

[B63] Kloner R. A. (2013). Current state of clinical translation of cardioprotective agents for acute myocardial infarction. Circ. Res. 113, 451–463. 10.1161/CIRCRESAHA.112.300627 23908332

[B64] Kobayashi T. Hirano K. Yamamoto T. Hasegawa G. Hatakeyama K. Suematsu M. (2002). The protective role of Kupffer cells in the ischemia-reperfused rat liver. Arch. Histol. Cytol. 65, 251–261. 10.1679/aohc.65.251 12389664

[B65] Koh E. J. Yoon S. J. Lee S. M. (2013). Losartan protects liver against ischaemia/reperfusion injury through PPAR-gamma activation and receptor for advanced glycation end-products down-regulation. Br. J. Pharmacol. 169, 1404–1416. 10.1111/bph.12229 23647130 PMC3831716

[B66] Kuboki S. Shin T. Huber N. Eismann T. Galloway E. Schuster R. (2008). Peroxisome proliferator-activated receptor-gamma protects against hepatic ischemia/reperfusion injury in mice. Hepatology 47, 215–224. 10.1002/hep.21963 18085707

[B67] Kurose I. Wolf R. Grisham M. B. Granger D. N. (1994). Modulation of ischemia/reperfusion-induced microvascular dysfunction by nitric oxide. Circ. Res. 74, 376–382. 10.1161/01.res.74.3.376 8118946

[B68] Lin L. Wang X. Yu Z. (2016). Ischemia-reperfusion injury in the brain: Mechanisms and potential therapeutic strategies. Biochem. Pharmacol. (Los Angel) 5. 10.4172/2167-0501.1000213 PMC5991620 29888120

[B69] Lee J. H. Amarsanaa K. Wu J. Jeon S. C. Cui Y. Jung S. G. (2018). Nobiletin attenuates neurotoxic mitochondrial calcium overload through K(+) influx and DeltaPsim across mitochondrial inner membrane. Korean J. Physiol. Pharmacol. 22, 311–319. 10.4196/kjpp.2018.22.3.311 29719453 PMC5928344

[B70] Lefebvre A. M. Chen I. Desreumaux P. Najib J. Fruchart J. C. Geboes K. (1998). Activation of the peroxisome proliferator-activated receptor gamma promotes the development of colon tumors in C57BL/6J-APCMin/+ mice. Nat. Med. 4, 1053–1057. 10.1038/2036 9734399

[B71] Li D. Chen K. Sinha N. Zhang X. Wang Y. Sinha A. K. (2005). The effects of PPAR-gamma ligand pioglitazone on platelet aggregation and arterial thrombus formation. Cardiovasc. Res. 65, 907–912. 10.1016/j.cardiores.2004.11.027 15721871

[B72] Li J. Xiang X. Gong X. Shi Y. Yang J. Xu Z. (2017). Cilostazol protects mice against myocardium ischemic/reperfusion injury by activating a PPARgamma/JAK2/STAT3 pathway. Biomed. Pharmacother. 94, 995–1001. 10.1016/j.biopha.2017.07.143 28810537

[B73] Li X. Kimura H. Hirota K. Sugimoto H. Kimura N. Takahashi N. (2007). Hypoxia reduces the expression and anti-inflammatory effects of peroxisome proliferator-activated receptor-gamma in human proximal renal tubular cells. Nephrol. Dial. Transpl. 22, 1041–1051. 10.1093/ndt/gfl766 17255125

[B74] Liang X. H. Jackson S. Seaman M. Brown K. Kempkes B. Hibshoosh H. (1999). Induction of autophagy and inhibition of tumorigenesis by beclin 1. Nature 402, 672–676. 10.1038/45257 10604474

[B75] Lin F. Xu L. Huang M. Deng B. Zhang W. Zeng Z. (2020). Beta-sitosterol protects against myocardial ischemia/reperfusion injury via targeting PPARgamma/NF-kappaB signalling. Evid. Based Complement. Alternat Med. 2020, 2679409. 10.1155/2020/2679409 32308701 PMC7142345

[B76] Lin T. N. Cheung W. M. Wu J. S. Chen J. J. Lin H. Chen J-J. (2006). 15D-prostaglandin J2 protects brain from ischemia-reperfusion injury. Arterioscler Thromb. Vasc. Biol. 26, 481–487. 10.1161/01.ATV.0000201933.53964.5b 16385084

[B77] Linares I. Farrokhi K. Echeverri J. Kaths J. M. Kollmann D. Hamar M. (2018). PPAR-gamma activation is associated with reduced liver ischemia-reperfusion injury and altered tissue-resident macrophages polarization in a mouse model. PLoS One 13, e195212. 10.1371/journal.pone.0195212 PMC5884549 29617419

[B78] Lipton S. A. (2006). NMDA receptors, glial cells, and clinical medicine. Neuron 50 (1), 9–11. 10.1016/j.neuron.2006.03.026 16600850

[B79] Liu C. Ding R. Huang W. Miao L. Li J. Li Y. (2020). Sevoflurane protects against intestinal ischemia-reperfusion injury by activating peroxisome proliferator-activated receptor gamma/nuclear factor-κb pathway in rats. Pharmacology 105 (3-4), 231–242. 10.1159/000503727 31655824

[B80] Liu X. Yu H. Yang L. Li C. Li L. (2012). 15-Deoxy-Delta(12,14)-prostaglandin J(2) attenuates the biological activities of monocyte/macrophage cell lines. Eur. J. Cel Biol 91, 654–661. 10.1016/j.ejcb.2012.03.004 22560326

[B81] Liu X. Yu Z. Huang X. Gao Y. Wang X. Gu J. (2016). Peroxisome proliferator-activated receptor gamma (PPARgamma) mediates the protective effect of quercetin against myocardial ischemia-reperfusion injury via suppressing the NF-kappaB pathway. Am. J. Transl Res. 8, 5169–5186. 28077993 PMC5209473

[B82] Liu Y. Zhang W. Cheng Y. Miao C. Gong J. Wang M. (2018). Activation of PPARgamma by Curcumin protects mice from ischemia/reperfusion injury induced by orthotopic liver transplantation via modulating polarization of Kupffer cells. Int. Immunopharmacol. 62, 270–276. 10.1016/j.intimp.2018.07.013 30036770

[B83] Ma S. Wang Y. Chen Y. Cao F. (2015). The role of the autophagy in myocardial ischemia/reperfusion injury. Biochim. Biophys. Acta 1852, 271–276. 10.1016/j.bbadis.2014.05.010 24859226

[B84] Magadum A. Engel F. B. (2018). PPARβ/δ: linking metabolism to regeneration. Int. J. Mol. Sci. 19 (7). 10.3390/ijms19072013 PMC6073704 29996502

[B85] Mahmoud-Awny M. Attia A. S. Abd-Ellah M. F. El-Abhar H. S. (2015). Mangiferin mitigates gastric ulcer in ischemia/reperfused rats: involvement of PPAR-γ, NF-κB and Nrf2/HO-1 signaling pathways. PLoS One 10 (7), e0132497. 10.1371/journal.pone.0132497 26196679 PMC4509761

[B86] Matsui Y. Takagi H. Qu X. Abdellatif M. Sakoda H. Asano T. (2007). Distinct roles of autophagy in the heart during ischemia and reperfusion: roles of AMP-activated protein kinase and Beclin 1 in mediating autophagy. Circ. Res. 100, 914–922. 10.1161/01.RES.0000261924.76669.36 17332429

[B87] Mohey V. Singh M. Puri N. Kaur T. Pathak D. Singh A. P. (2016). Sildenafil obviates ischemia-reperfusion injury-induced acute kidney injury through peroxisome proliferator-activated receptor gamma agonism in rats. J. Surg. Res. 201, 69–75. 10.1016/j.jss.2015.09.035 26850186

[B88] Morrison A. Yan X. Tong C. Li J. (2011). Acute rosiglitazone treatment is cardioprotective against ischemia-reperfusion injury by modulating AMPK, Akt, and JNK signaling in nondiabetic mice. Am. J. Physiol. Heart Circ. Physiol. 301, H895–H902. 10.1152/ajpheart.00137.2011 21666107

[B89] Murphy G. J. Holder J. C. (2000). PPAR-gamma agonists: therapeutic role in diabetes, inflammation and cancer. Trends Pharmacol. Sci. 21, 469–474. 10.1016/s0165-6147(00)01559-5 11121836

[B90] Nakajima A. Wada K. Miki H. Kubota N. Nakajima N. Terauchi Y. (2001). Endogenous PPAR gamma mediates anti-inflammatory activity in murine ischemia-reperfusion injury. Gastroenterology 120, 460–469. 10.1053/gast.2001.21191 11159886

[B91] Nissen S. E. Nicholls S. J. Wolski K. Howey D. C. McErlean E. Wang M. D. (2007). Effects of a potent and selective PPAR-alpha agonist in patients with atherogenic dyslipidemia or hypercholesterolemia: two randomized controlled trials. JAMA 297 (12), 1362–1373. 10.1001/jama.297.12.1362 17384435

[B92] Nissen S. E. Wolski K. (2007). Effect of rosiglitazone on the risk of myocardial infarction and death from cardiovascular causes. N. Engl. J. Med. 356, 2457–2471. 10.1056/NEJMoa072761 17517853

[B93] Okada Y. Marchevsky A. M. Zuo X. J. Pass J. A. Kass R. M. Matloff J. M. (1997). Accumulation of platelets in rat syngeneic lung transplants: a potential factor responsible for preservation-reperfusion injury. Transplantation 64, 801–806. 10.1097/00007890-199709270-00002 9326401

[B94] Pelin M. Fusco L. Martin C. Sosa S. Frontinan-Rubio J. González-Domínguez J. M. (2018). Graphene and graphene oxide induce ROS production in human HaCaT skin keratinocytes: the role of xanthine oxidase and NADH dehydrogenase. Nanoscale 10, 11820–11830. 10.1039/c8nr02933d 29920573

[B95] Phull A. R. Nasir B. Haq I. U. Kim S. J. (2018). Oxidative stress, consequences and ROS mediated cellular signaling in rheumatoid arthritis. Chem. Biol. Interact 281, 121–136. 10.1016/j.cbi.2017.12.024 29258867

[B96] Piche M. E. Laberge A. S. Brassard P. Arsenault B. J. Bertrand O. F. Després J. D. (2018). Rosiglitazone lowers resting and blood pressure response to exercise in men with type 2 diabetes: a 1-year randomized study. Diabetes Obes. Metab. 20, 1740–1750. 10.1111/dom.13293 29573098

[B97] Plutzky J. (2012). PPARs and cardiovascular disease risk reduction in patients with type 2 diabetes. Medscapes CME & EDUCATION.

[B98] Posada-Duque R. A. Barreto G. E. Cardona-Gomez G. P. (2014). Protection after stroke: cellular effectors of neurovascular unit integrity. Front. Cel Neurosci 8, 231. 10.3389/fncel.2014.00231 PMC4132372 25177270

[B99] Pruimboom-Brees I. M. Francone O. Pettersen J. C. Kerlin R. L. Will Y. Amacher D. E. (2012). The development of subcutaneous sarcomas in rodents exposed to peroxisome proliferators agonists: hypothetical mechanisms of action and de-risking attitude. Toxicol. Pathol. 40, 810–818. 10.1177/0192623312441406 22504321

[B100] Puyal J. Ginet V. Grishchuk Y. Truttmann A. C. Clarke P. G. (2012). Neuronal autophagy as a mediator of life and death: contrasting roles in chronic neurodegenerative and acute neural disorders. Neuroscientist 18 (3), 224–236. 10.1177/1073858411404948 21525331

[B101] Puyal J. Clarke P. G. (2009). Targeting autophagy to prevent neonatal stroke damage. Autophagy 5, 1060–1061. 10.4161/auto.5.7.9728 19713756

[B102] Qi D. S. Tao J. H. Zhang L. Q. Li M. Wang M. Qu R. (2016). Neuroprotection of Cilostazol against ischemia/reperfusion-induced cognitive deficits through inhibiting JNK3/caspase-3 by enhancing Akt1. Brain Res. 1653, 67–74. 10.1016/j.brainres.2016.10.017 27769787

[B103] Qian J. Chen H. Birnbaum Y. Nanhwan M. K. Bajaj M. Ye Y. (2016). Aleglitazar, a balanced dual PPARalpha and -gamma agonist, protects the heart against Ischemia-Reperfusion injury. Cardiovasc. Drugs Ther. 30, 129–141. 10.1007/s10557-016-6650-9 26861490

[B104] Qin H. Tan W. Zhang Z. Bao L. Shen H. Wang F. (2015). 15D-prostaglandin J2 protects cortical neurons against oxygen-glucose deprivation/reoxygenation injury: involvement of inhibiting autophagy through upregulation of Bcl-2. Cell Mol Neurobiol 35, 303–312. 10.1007/s10571-014-0125-y 25349027 PMC11486323

[B105] Ragab D. Abdallah D. M. El-Abhar H. S. (2014). Cilostazol renoprotective effect: modulation of PPAR-gamma, NGAL, KIM-1 and IL-18 underlies its novel effect in a model of ischemia-reperfusion. PLoS One 9, e95313. 10.1371/journal.pone.0095313 24816434 PMC4015937

[B106] Rani N. Arya D. S. (2020). Chrysin rescues rat myocardium from ischemia-reperfusion injury via PPAR-gamma/Nrf2 activation. Eur. J. Pharmacol. 883, 173389. 10.1016/j.ejphar.2020.173389 32707190

[B107] Raskin P. Rappaport E. B. Cole S. T. Yan Y. Patwardhan R. Freed M. I. (2000). Rosiglitazone short-term monotherapy lowers fasting and post-prandial glucose in patients with type II diabetes. Diabetologia 43, 278–284. 10.1007/s001250050045 10768088

[B108] Rautou P. E. Mansouri A. Lebrec D. Durand F. Valla D. Moreau R. (2010). Autophagy in liver diseases. J. Hepatol. 53 (6), 1123–1134. 10.1016/j.jhep.2010.07.006 20810185

[B109] Raz L. Zhang Q. G. Zhou C. F. Han D. Gulati P. Yang L. C. (2010). Role of Rac1 GTPase in NADPH oxidase activation and cognitive impairment following cerebral ischemia in the rat. PLoS One 5, e12606. 10.1371/journal.pone.0012606 20830300 PMC2935374

[B110] Reel B. Guzeloglu M. Bagriyanik A. Atmaca S. Aykut K. Albayrak G. (2013). The effects of PPAR-gamma agonist pioglitazone on renal ischemia/reperfusion injury in rats. J. Surg. Res. 182, 176–184. 10.1016/j.jss.2012.08.020 22981741

[B111] Refaie M. M. M. El-Hussieny M. (2018). Protective effect of pioglitazone on ovarian ischemia reperfusion injury of female rats via modulation of peroxisome proliferator activated receptor gamma and heme-oxygenase 1. Int. Immunopharmacol. 62, 7–14. 10.1016/j.intimp.2018.06.037 29966944

[B112] Riess M. L. Elorbany R. Weihrauch D. Stowe D. F. Camara A. (2020). PPARgamma-Independent side effects of thiazolidinediones on mitochondrial redox state in rat isolated hearts. Cells 9, (1):252. 10.3390/cells9010252 PMC7017211 31968546

[B113] Rogue A. Spire C. Brun M. Claude N. Guillouzo A. (2010). Gene expression changes induced by PPAR gamma agonists in animal and human liver. PPAR Res. 2010, 325183. 10.1155/2010/325183 20981297 PMC2963138

[B114] Rosenberg G. A. (2012). Neurological diseases in relation to the blood-brain barrier. J. Cereb. Blood Flow Metab. 32, 1139–1151. 10.1038/jcbfm.2011.197 22252235 PMC3390801

[B115] Rossi D. J. Oshima T. Attwell D. (2000). Glutamate release in severe brain ischaemia is mainly by reversed uptake. Nature 403 (6767), 316–321. 10.1038/35002090 10659851

[B116] Ruiz-Miyazawa K. W. Staurengo-Ferrari L. Pinho-Ribeiro F. A. Fattori V. Zaninelli T. H. Badaro-Garcia S. (2018). 15D-PGJ2-loaded nanocapsules ameliorate experimental gout arthritis by reducing pain and inflammation in a PPAR-gamma-sensitive manner in mice. Sci. Rep. 8, 13979. 10.1038/s41598-018-32334-0 30228306 PMC6143605

[B117] Sato H. Sugai H. Kurosaki H. Ishikawa M. Funaki A. Kimura Y. (2013). The effect of sex hormones on peroxisome proliferator-activated receptor gamma expression and activity in mature adipocytes. Biol. Pharm. Bull. 36, 564–573. 10.1248/bpb.b12-00868 23546292

[B118] Sciarretta S. Maejima Y. Zablocki D. Sadoshima J. (2018). The role of autophagy in the heart. Annu. Rev. Physiol. 80, 1–26. 10.1146/annurev-physiol-021317-121427 29068766

[B119] Shao Z. Q. Liu Z. J. (2015). Neuroinflammation and neuronal autophagic death were suppressed via Rosiglitazone treatment: new evidence on neuroprotection in a rat model of global cerebral ischemia. J. Neurol. Sci. 349, 65–71. 10.1016/j.jns.2014.12.027 25623802

[B120] Shen Y. Wu H. Wang C. Shao H. Huang H. Jing H. (2010). Simvastatin attenuates cardiopulmonary bypass-induced myocardial inflammatory injury in rats by activating peroxisome proliferator-activated receptor gamma. Eur. J. Pharmacol. 649, 255–262. 10.1016/j.ejphar.2010.08.058 20858481

[B121] Sheng R. Zhang L. S. Han R. Liu X. Q. Gao B. Qin Z-H. (2010). Autophagy activation is associated with neuroprotection in a rat model of focal cerebral ischemic preconditioning. Autophagy 6, 482–494. 10.4161/auto.6.4.11737 20400854

[B122] Shimazaki N. Togashi N. Hanai M. Isoyama T. Wada K. Fujita T. (2008). Anti-tumour activity of CS-7017, a selective peroxisome proliferator-activated receptor gamma agonist of thiazolidinedione class, in human tumour xenografts and a syngeneic tumour implant model. Eur. J. Cancer 44, 1734–1743. 10.1016/j.ejca.2008.04.016 18511262

[B123] Shimazu T. Inoue I. Araki N. Asano Y. Sawada M. Furuya D. (2005). A peroxisome proliferator-activated receptor-gamma agonist reduces infarct size in transient but not in permanent ischemia. Stroke 36, 353–359. 10.1161/01.STR.0000152271.21943.a2 15618443

[B124] Simon R. P. Swan J. H. Griffiths T. Meldrum B. S. (1984). Blockade of N-methyl-D-aspartate receptors may protect against ischemic damage in the brain. Science 226 (4676), 850–852. 10.1126/science.6093256 6093256

[B125] Singh A. P. Singh N. Bedi P. M. (2016). Pioglitazone ameliorates renal ischemia reperfusion injury through NMDA receptor antagonism in rats. Mol. Cel Biochem 417, 111–118. 10.1007/s11010-016-2718-x 27206738

[B126] Singh A. P. Singh N. Pathak D. Bedi P. (2019). Estradiol attenuates ischemia reperfusion-induced acute kidney injury through PPAR-gamma stimulated eNOS activation in rats. Mol. Cel Biochem 453, 1–9. 10.1007/s11010-018-3427-4 30194582

[B127] Sivarajah A. Chatterjee P. K. Patel N. S. Todorovic Z. Hattori Y. Brown P. A. (2003). Agonists of peroxisome-proliferator activated receptor-gamma reduce renal ischemia/reperfusion injury. Am. J. Nephrol. 23, 267–276. 10.1159/000072088 12840602

[B128] Sohn K. A. Cruciani-Guglielmacci C. Kassis N. Clement L. Ouali F. Caüzac M. (2009). S26948, a new specific peroxisome proliferator activated receptor gamma modulator improved *in vivo* hepatic insulin sensitivity in 48 h lipid infused rats. Eur. J. Pharmacol. 608, 104–111. 10.1016/j.ejphar.2009.02.033 19250932

[B129] Son N. H. Yu S. Tuinei J. Arai K. Hamai H. Homma S. (2010). PPARγ-induced cardiolipotoxicity in mice is ameliorated by PPARα deficiency despite increases in fatty acid oxidation. J. Clin. Invest. 120 (10), 3443–3454. 10.1172/JCI40905 20852389 PMC2947216

[B130] Tokutome M. Matoba T. Nakano Y. Okahara A. Fujiwara M. Koga J-I. (2019). Peroxisome proliferator-activated receptor-gamma targeting nanomedicine promotes cardiac healing after acute myocardial infarction by skewing monocyte/macrophage polarization in preclinical animal models. Cardiovasc. Res. 115, 419–431. 10.1093/cvr/cvy200 30084995

[B131] Vitale S. G. Lagana A. S. Nigro A. La Rosa V. L. Rossetti P. Rossetti P. (2016). Peroxisome Proliferator-Activated receptor modulation during metabolic diseases and cancers: master and minions. PPAR Res. 2016, 6517313. 10.1155/2016/6517313 28115924 PMC5225385

[B132] Wang H. Zhu Q. W. Ye P. Li Z. B. Li Y. Cao Z. L. (2012). Pioglitazone attenuates myocardial ischemia-reperfusion injury via up-regulation of ERK and COX-2. Biosci. Trends 6, 325–332. 23337792

[B133] Wang L. Lin R. Guo L. Hong M. (2018). Rosuvastatin relieves myocardial ischemia/reperfusion injury by upregulating PPARgamma and UCP2. Mol. Med. Rep. 18, 789–798. 10.3892/mmr.2018.9062 29845235 PMC6059708

[B134] Wang X. Li R. Wang X. Fu Q. Ma S. (2015). Umbelliferone ameliorates cerebral ischemia-reperfusion injury via upregulating the PPAR gamma expression and suppressing TXNIP/NLRP3 inflammasome. Neurosci. Lett. 600, 182–187. 10.1016/j.neulet.2015.06.016 26071904

[B135] Watanabe K. Shishido T. Otaki Y. Watanabe T. Sugai T. Toshima T. (2019). Increased plasma xanthine oxidoreductase activity deteriorates coronary artery spasm. Heart Vessels 34, 1–8. 10.1007/s00380-018-1207-4 29936631

[B136] Wei W. Wan Y. (2011). Thiazolidinediones on PPARgamma: the roles in bone remodeling. PPAR Res. 2011, 867180. 10.1155/2011/867180 22135675 PMC3205770

[B137] Willson T. M. Brown P. J. Sternbach D. D. Henke B. R. (2000). The PPARs: from orphan receptors to drug discovery. J. Med. Chem. 43, 527–550. 10.1021/jm990554g 10691680

[B138] Wu G. Wu J. Jiao Y. Wang L. Wang F. Zhang Y. (2015). Rosiglitazone infusion therapy following minimally invasive surgery for intracerebral hemorrhage evacuation decreases matrix metalloproteinase-9 and blood-brain barrier disruption in rabbits. BMC Neurol. 15, 37. 10.1186/s12883-015-0287-3 26021445 PMC4472168

[B139] Wu L. Yan C. Czader M. Foreman O. Blum J. S. Kapur R. (2012). Inhibition of PPARgamma in myeloid-lineage cells induces systemic inflammation, immunosuppression, and tumorigenesis. Blood 119, 115–126. 10.1182/blood-2011-06-363093 22053106 PMC3251224

[B140] Wu X. J. Sun X. H. Wang S. W. Chen J. L. Bi Y. H. Jiang D-X. (2018). Mifepristone alleviates cerebral ischemia-reperfusion injury in rats by stimulating PPAR gamma. Eur. Rev. Med. Pharmacol. Sci. 22, 5688–5696. 10.26355/eurrev_201809_15836 30229846

[B141] Xi X. Zou C. Ye Z. Huang Y. Chen T. Hu H. (2019). Pioglitazone protects tubular cells against hypoxia/reoxygenation injury through enhancing autophagy via AMPK-mTOR signaling pathway. Eur. J. Pharmacol. 863, 172695. 10.1016/j.ejphar.2019.172695 31560869

[B142] Xia P. Pan Y. Zhang F. Wang N. Wang E. Guo Q. (2018). Pioglitazone confers neuroprotection against ischemia-induced pyroptosis due to its inhibitory effects on HMGB-1/RAGE and Rac1/ROS pathway by activating PPAR-ɤ. Cell Physiol Biochem 45, 2351–2368. 10.1159/000488183 29554649

[B143] Xiong D. Deng Y. Huang B. Yin C. Liu B. Shi J. (2016). Icariin attenuates cerebral ischemia-reperfusion injury through inhibition of inflammatory response mediated by NF-kappaB, PPARalpha and PPARgamma in rats. Int. Immunopharmacol 30, 157–162. 10.1016/j.intimp.2015.11.035 26679678

[B144] Xu F. Gu J. H. Qin Z. H. (2012). Neuronal autophagy in cerebral ischemia. Neurosci. Bull. 28, 658–666. 10.1007/s12264-012-1268-9 22968594 PMC5561920

[B145] Xu F. Li J. Ni W. Shen Y. W. Zhang X. P. (2013). Peroxisome proliferator-activated receptor-gamma agonist 15d-prostaglandin J2 mediates neuronal autophagy after cerebral ischemia-reperfusion injury. PLoS One 8, e55080. 10.1371/journal.pone.0055080 23372817 PMC3555818

[B146] Xu Y. Gen M. Lu L. Fox J. Weiss S. O. Brown R. D. (2005). PPAR-gamma activation fails to provide myocardial protection in ischemia and reperfusion in pigs. Am. J. Physiol. Heart Circ. Physiol. 288, H1314–H1323. 10.1152/ajpheart.00618.2004 15528232 PMC3633522

[B147] Xu Y. Huo Y. Toufektsian M. C. Ramos S. I. Ma Y. Tejani A. D. (2006). Activated platelets contribute importantly to myocardial reperfusion injury. Am. J. Physiol. Heart Circ. Physiol. 290, H692–H699. 10.1152/ajpheart.00634.2005 16199480

[B148] Xu Y. Lu L. Greyson C. Lee J. Gen M. Kinugawa K. (2003). Deleterious effects of acute treatment with a peroxisome proliferator-activated receptor-gamma activator in myocardial ischemia and reperfusion in pigs. Diabetes 52, 1187–1194. 10.2337/diabetes.52.5.1187 12716751 PMC3633427

[B149] Xu Y. Yao J. Zou C. Zhang H. Zhang S. Liu J. (2017). Asiatic acid protects against hepatic ischemia/reperfusion injury by inactivation of Kupffer cells via PPARgamma/NLRP3 inflammasome signaling pathway. Oncotarget 8, 86339–86355. 10.18632/oncotarget.21151 29156799 PMC5689689

[B150] Yang Z. Klionsky D. J. (2010). Mammalian autophagy: core molecular machinery and signaling regulation. Curr. Opin. Cel Biol 22, 124–131. 10.1016/j.ceb.2009.11.014 PMC2854249 20034776

[B151] Yasuda S. Kobayashi H. Iwasa M. Kawamura I. Sumi S. Narentuoya B. (2009). Antidiabetic drug pioglitazone protects the heart via activation of PPAR-gamma receptors, PI3-kinase, Akt, and eNOS pathway in a rabbit model of myocardial infarction. Am. J. Physiol. Heart Circ. Physiol. 296, H1558–H1565. 10.1152/ajpheart.00712.2008 19286954

[B152] Ye Y. Lin Y. Manickavasagam S. Perez-Polo J. R. Tieu B. C. Birnbaum Y. (2008). Pioglitazone protects the myocardium against ischemia-reperfusion injury in eNOS and iNOS knockout mice. Am. J. Physiol. Heart Circ. Physiol. 295, H2436–H2446. 10.1152/ajpheart.00690.2008 18931027

[B153] Ysebaert D. K. De Greef K. E. De Beuf A. Van Rompay A. R. Vercauteren S. Persy V. P. (2004). T cells as mediators in renal ischemia/reperfusion injury. Kidney Int. 66, 491–496. 10.1111/j.1523-1755.2004.761_4.x 15253695

[B154] Yu W. Xu M. Zhang T. Zhang Q. Zou C. (2019). Mst1 promotes cardiac ischemia-reperfusion injury by inhibiting the ERK-CREB pathway and repressing FUNDC1-mediated mitophagy. J. Physiol. Sci. 69, 113–127. 10.1007/s12576-018-0627-3 29961191 PMC10717665

[B155] Zhang H. Zhang A. Kohan D. E. Nelson R. D. Gonzalez F. J. Yang T. (2005). Collecting duct-specific deletion of peroxisome proliferator-activated receptor gamma blocks thiazolidinedione-induced fluid retention. Proc. Natl. Acad. Sci. U S A 102, 9406–9411. 10.1073/pnas.0501744102 15956187 PMC1166599

[B156] Zhang Q. G. Wang R. Han D. Dong Y. Brann D. W. (2009). Role of Rac1 GTPase in JNK signaling and delayed neuronal cell death following global cerebral ischemia. Brain Res. 1265, 138–147. 10.1016/j.brainres.2009.01.033 19368836 PMC3801190

[B157] Zhang Q. Shao M. Zhang X. Wang Q. Guo D. Yang X. (2018). The effect of Chinese medicine on lipid and glucose metabolism in acute myocardial infarction through PPARgamma pathway. Front. Pharmacol. 9, 1209. 10.3389/fphar.2018.01209 30405421 PMC6207917

[B158] Zhang X. Y. Xiao Y. Q. Zhang Y. Ye W. (2013). Protective effect of pioglitazone on retinal ischemia/reperfusion injury in rats. Invest. Ophthalmol. Vis. Sci. 54 (6), 3912–3921. 10.1167/iovs.09-4509 23557740

[B159] Zhang Y. L. Wang R. B. Li W. Y. Xia F. Z. Liu L. (2017). Pioglitazone ameliorates retinal ischemia/reperfusion injury via suppressing NLRP3 inflammasome activities. Int. J. Ophthalmol. 10 (12), 1812–1818. 10.18240/ijo.2017.12.04 29259897 PMC5733506

[B160] Zhou H. Li D. Zhu P. Hu S. Hu N. Ma S. (2017). Melatonin suppresses platelet activation and function against cardiac ischemia/reperfusion injury via PPARgamma/FUNDC1/mitophagy pathways. J. Pineal Res. 63, [Epub ahead of print]. 10.1111/jpi.12438 28749565

[B161] Zhou H. Sun J. Zhong W. Pan X. Liu C. Cheng F. (2020). Dexmedetomidine preconditioning alleviated murine liver ischemia and reperfusion injury by promoting macrophage M2 activation via PPARgamma/STAT3 signaling. Int. Immunopharmacol. 82, 106363. 10.1016/j.intimp.2020.106363 32145512

[B162] Zhou Y. Jia S. Wang C. Chen Z. Chi Y. (2013). FAM3A is a target gene of peroxisome proliferator-activated receptor gamma. Biochim. Biophys. Acta 1830, 4160–4170. 10.1016/j.bbagen.2013.03.029 23562554

[B163] Zou C. Hu H. Xi X. Shi Z. Wang G. Li J. (2013). Pioglitazone protects against renal ischemia-reperfusion injury by enhancing antioxidant capacity. J. Surg. Res. 184, 1092–1095. 10.1016/j.jss.2013.03.027 23545406

[B164] Zou C. Zhou Z. Tu Y. Wang W. Chen T. Hu H. (2019). Pioglitazone attenuates reoxygenation injury in renal tubular NRK-52E cells exposed to high glucose via inhibiting oxidative stress and endoplasmic reticulum stress. Front. Pharmacol. 10, 1607. 10.3389/fphar.2019.01607 32038263 PMC6989595

[B165] Zuo Y. Huang L. Enkhjargal B. Xu W. Umut O. Travis Z. D. (2019). Activation of retinoid X receptor by bexarotene attenuates neuroinflammation via PPARgamma/SIRT6/FoxO3a pathway after subarachnoid hemorrhage in rats. J. Neuroinflammation 16, 47. 10.1186/s12974-019-1432-5 30791908 PMC6385420

